# Barriers and facilitators of HPV vaccination in sub-saharan Africa: a systematic review

**DOI:** 10.1186/s12889-023-15842-1

**Published:** 2023-05-26

**Authors:** Jean-Marc Kutz, Pia Rausche, Tarik Gheit, Dewi Ismajani Puradiredja, Daniela Fusco

**Affiliations:** 1 Department of Infectious Diseases Epidemiology, Bernhard Nocht Institute for Tropical Medicine (BNITM), Hamburg, Germany; 2 German Center for Infection Research (DZIF), Hamburg-Borstel-Lübeck-Riems, Germany; 3 International Agency for Research on Cancer (IARC), Lyon, France

**Keywords:** Human papillomavirus viruses [Mesh], Papillomavirus Vaccines [Mesh], Sub-Saharan Africa [Mesh], Uterine cervical neoplasms [Mesh]

## Abstract

**Background:**

Human Papilloma Virus (HPV) is the most common sexually transmitted infection worldwide. Globally, both men and women have a 50% risk of being infected at least once in their life. HPV prevalence is among the highest in sub-Saharan Africa (SSA), at an average of 24%. HPV causes different types of cancers, including cervical cancer (CC), which is the leading cause of cancer deaths among women in SSA. HPV-vaccination has been proven to be effective in reducing HPV induced cancers. SSA countries are delayed in reaching the WHO’s target of fully vaccinating 90% of girls within the age of 15 by 2030. Our systematic review aims to identify barriers and facilitators of HPV-vaccination in SSA to inform national implementation strategies in the region.

**Methods:**

This is a mixed method systematic review based on the PRISMA statement and The Joanna Briggs Institute Reviewers’ Manual. Search strategies were adapted to each selected database: PubMed/MEDLINE, Livivo, Google Scholar, Science Direct, and African Journals Online for papers published in English, Italian, German, French and Spanish between 1 December 2011 and 31 December 2021. Zotero and Rayyan were the software used for data management. The appraisal was conducted by three independent reviewers.

**Results:**

A total of 20 articles were selected for appraisal from an initial 536 articles. Barriers included: limited health system capacities, socio-economic status, stigma, fear and costs of vaccines, negative experience with vaccinations, COVID-19 pandemic, lack of correct information, health education (HE) and consent. Additionally, we found that boys are scarcely considered for HPV-vaccination by parents and stakeholders. Facilitators included: information and knowledge, policy implementation, positive experience with vaccinations, HE, stakeholders’ engagement, women’s empowerment, community engagement, seasonality, and target-oriented vaccination campaigns.

**Conclusions:**

This review synthesizes barriers and facilitators of HPV-vaccinations in SSA. Addressing these can contribute to the implementation of more effective HPV immunization programs targeted at eliminating CC in line with the WHO 90/70/90 strategy.

**Registration and funding:**

Protocol ID: CRD42022338609 registered in the International Prospective Register of Systematic Reviews (PROSPERO). Partial funds: German Centre for Infection research (DZIF) project NAMASTE: 8,008,803,819.

**Supplementary Information:**

The online version contains supplementary material available at 10.1186/s12889-023-15842-1.

## Contributions to the literature

Our review synthesizes factors influencing the successful implementation of HPV vaccination programs in SSA in order to improve roll-out and coverage.

Our review provides an overview and contextualizes the barriers and facilitators of HPV vaccination implementation in 12 countries in SSA. These span the health care system ranging from personal experiences to economic, social, and political factors.

Lessons learnt from past experiences of HPV vaccination in SSA as identified by our review, can contribute to more effective national HPV vaccination programs targeted at eliminating CC and implementing the WHO 90/70/90 triple intervention strategy.

**Supplementary Information:**

The online version contains supplementary material available at 10.1186/s12889-023-15842-1.

## Background

Human Papilloma Virus (HPV) infects basal keratinocytes of the mucosal and cutaneous epithelia and is the cause of common dermatologic diseases as well as of various other types of cancers [1]. Globally, it represents the most common sexually transmitted infection (STI) [2]. The infection is often naturally cleared by the host immune system within one or two years, otherwise it can persist silently in infected individuals, with varying pathological effects, such as cancer onset and progression [1].

There are more than 200 types of HPV that are classified into five major genera: alpha, beta, gamma, mu, and nu [3]. HPV types are commonly divided into high (HR - carcinogenic) or low-risk (LR- non-carcinogenic) types [1]. CC is the most frequent type of cancer associated with HPV infection and is almost always associated with HR-HPV [4]. Other types of cancers, including cancers of the anus, penis, vagina, vulva, and the oropharynx, have been linked to HPV infection [5].

The infections are often transmitted through micro-wounds incurred during sexual intercourse or through other types of skin-to-skin contact [6]. Both heterosexual and homosexual HPV transmission is possible through penetrative and non-penetrative sexual contact [7]. HPV infection frequency varies according to the anatomical site: in non-cervical sites, a higher prevalence is shown in the anogenital than in the oral region. Both men and women worldwide have an estimated 50% risk of getting infected at least once in a lifetime [8]. HPV prevalence is with an estimated average of 24% among the highest in sub-Saharan Africa (SSA) [9].

Vaccines against HPV became first available in 2006 and protect against the strains most likely to cause genital warts or CC [10]. Currently there are six licensed HPV vaccines: three bivalent, two quadrivalent, and one nonavalent [11]; four of these are WHO prequalified [12]. Real-world data indicates that HPV vaccination cuts cervical cancer cases by about 90% [13]. Specifically, the bivalent and quadrivalent vaccines target the two main HR genotypes (16 and 18), which cause approximately 70% of CC worldwide, while the nonavalent targets nine different HR genotypes, which cause approximately 90% of cervical CC worldwide. The quadrivalent and nonavalent vaccines also protect against two LR genotypes (6 and 11), responsible for approximately 90% of genital warts [14–16]. The World Health Organization (WHO) recommended the use of a two doses regimen for girls aged nine to 13 years in 2009. Nevertheless, in 2016, it was estimated that HPV immunization programs reached only 12% of young adolescent girls worldwide [17]. HPV immunization strategies vary between different countries both in terms of immunization schedule and targeted risk groups. At the beginning of the roll-out, many national strategies targeted vaccination for girls aged 12–13 under the assumption that boys would be indirectly protected from HPV through “herd immunity” [18]. This approach, however, offers limited protection for men who have sex with men (MSM) [19]. As a result, many countries introduced vaccination for all adolescents regardless of their sex at birth [17]. The American Center of Disease Control (CDC) currently recommends a two (or three)-dose HPV vaccination regimen for boys and girls at age 11 or 12 as well as for everyone up until the age of 26 years if they had not been fully vaccinated at a younger age. The WHO recently revised the recommendations for girls aged 9–14 years to either a two-dose or a one-dose regimen [20] endorsing the high relevance of the vaccine in protecting against onset and progression of CC [21].

CC is a highly preventable cancer but still remains the main cause of cancer death in women in 36 low and middle-income countries (LMICs) [22–24]. In SSA, CC represents the second leading cause of cancer and the leading cause of cancer deaths in women [24]. CC can be prevented through primary (HPV vaccination), secondary (cervical screening and treatment of precancerous lesions) and tertiary (early diagnosis and treatment of cancer) prevention, which should be combined to reduce morbidity and mortality [25]. The lack of population-based screening programs has led to much higher CC incidence and mortality rates in LMICs than in high income countries. The implementation of population-based screening programs have been shown to be challenging in LMICs in general and in SSA in particular, due to financial, logistical, and socio-cultural factors [26].

In November 2020, the WHO launched the 90/70/90 triple intervention strategy - a global initiative to eliminate CC as a public health problem. The strategy aims to vaccinate at least 90% of girls against HPV by the age of 15 years, to screen 70% of women using a high-performance test by the age of 35 years and again by the age of 45, and to treat at least 90% of identified precancerous lesions and invasive cancers [27]. The WHO call for the elimination of CC using the 90/70/90 strategy is expected to be able to eliminate CC as a public health problem within a century with over 62 million cases averted by 2120 [27]. In the past, the high cost of the vaccine had hampered uptake in LMICs. GAVI commits to closing the equity gap by ensuring low sustainable prices. Still, nearly half of LMICs have been unable to introduce HPV vaccinations, as many countries cannot afford the negotiated $4,50 per dose [12, 28].

More than ten years of implementation programs have produced key lessons learned in terms of increasing vaccination coverage in many LMICs [29]. However, compared to other regions of the world, SSA has made limited progress in the implementation and performance of nationwide HPV vaccination programs [30, 31]. Rwanda was the first SSA country to introduce HPV vaccination in 2011 [32]. Subsequently, there has been a slow increase in the number of countries adopting the vaccine each year that peaked in 2019 with six additional SSA countries (The Gambia, Liberia, Côte d’Ivoire, Kenya, Malawi and Zambia) to follow suit [12, 17]. Without timely intervention, this will undermine the CC elimination efforts in this region.

Our systematic review aims to identify barriers and facilitators of HPV vaccination in SSA to inform pilot roll-out and national implementation strategies, that will contribute more effectively to the WHO goal of eliminating CC as public health problem by 2120.

## Methods

This systematic review uses the procedure of the “Integrated methodology” as described by “The Joanna Briggs Institute Reviewers’ Manual” in 2015 [33]. It combines quantitative and qualitative study results into a mixed method synthesis and refers to the Preferred Reporting Items for Systematic Reviews and Meta-Analyses (PRISMA) Statement [34]. The review protocol was registered in the International Prospective Register of Systematic Reviews (PROSPERO) with the ID CRD42022338609. A PRISMA checklist was used to assess compliance versus systematic reviews standardized methodologies. A meta-analysis was not conducted due to the heterogeneity of the data included.

### Searches

The literature search to identify target papers was performed using the syntax reported in the Table 1. The following databases were used for each respective syntax: PubMed/MEDLINE, Livivo, Google Scholar, Science Direct and African Journals Online (AJOL).

**Table 1:** Search syntax according to the listed databases

Database	Adapted search syntax
PubMed (MEDLINE)	((“**HPV**” OR “HPV16” OR “HPV18” OR “HPV6” OR “HPV 5” OR “HPV18” OR “HPV31” OR “HPV33” OR “HPV45” OR “HPV52” OR “Human papilloma Virus”) AND (“cover*” OR “prevent*” OR **“uptake”** OR **“status”** OR “access*” OR “program*” OR “strateg*” OR “guidelin*” OR “polic*”) AND (“vaccin*” OR “immun*” OR “Cervarix” OR “Gardasil” OR **“cervix cancer prevention”** OR **“cervix cancer control”**) AND (“barrier*” OR “block*” OR “limit*” OR “improv*” OR “factor*” OR “understand*” OR “analys*” OR “invest*” OR “willing*” OR **“knowledge”** OR **“awareness”** OR “accept*” OR **“hesitancy”** OR “challeng*”) AND (“Sub Saharan Africa” OR “SSA” OR “Angola” OR “Benin” OR “Botswana” OR “Burkina Faso” OR “Burundi” OR “Cameroon” OR “Cape Verde” OR “Central African Republic” OR “Chad” OR “Comoros” OR “Congo” OR “Côte d’Ivoire” OR “Djibouti” OR “Equatorial Guinea” OR “Eritrea” OR “Ethiopia” OR “Gabon” OR “Gambia” OR “Ghana” OR “Guinea” OR “Kenya” OR “Lesotho” OR “Liberia” OR “Madagascar” OR “Malawi” OR “Mali” OR “Mauritania” OR “Mauritius” OR “Mozambique” OR “Namibia” OR “Niger” OR “Nigeria” OR “Réunion” OR “Rwanda” OR “Sao Tome” and “Principe” OR “Senegal” OR “Seychelles” OR “Sierra Leone” OR “Somalia” OR “South Africa” OR “Sudan” OR “Swaziland” OR “Eswatini” OR “Tanzania” OR “Togo” OR “Uganda” OR “Western Sahara” OR “Zambia” OR “Zimbabwe”) NOT (“therapeutic*” OR “treatment*” OR " biomarker*” OR " **HIV** " OR “**cervical cancer screening**” OR “**respiratory papillomatosis**” OR “**chlamydia trachomatis**” OR “**trichomonas vaginalis**” OR “**vaccine efficiency**” OR “**immunology**” OR “**cost-effectiveness**” OR “**microbiome**” OR “**surfactant protein**” OR “**self-sampling**”))
Livivo	((**HPV** OR HPV16 OR HPV18 OR HPV6 OR HPV 5 OR HPV18 OR HPV31 OR HPV33 OR HPV45 OR HPV52 OR Human papilloma virus) AND (cover* OR prevent* OR **uptake** OR **status** OR access* OR program* OR strateg* OR guidelin* OR polic*) AND (vaccin* OR immun* OR Cervarix OR Gardasil OR **cervix cancer prevention** OR **cervix cancer control**) AND (barrier* OR block* OR limit* OR improv* OR factor* OR understand* OR analys* OR invest* OR willing* OR **knowledge** OR **awareness** OR accept* OR **hesitancy** OR challeng*) AND (Sub Saharan Africa OR SSA OR Angola OR Benin OR Botswana OR Burkina Faso OR Burundi OR Cameroon OR Cape Verde OR Central African Republic OR Chad OR Comoros OR Congo OR Côte d’Ivoire OR Djibouti OR Equatorial Guinea OR Eritrea OR Ethiopia OR Gabon OR Gambia OR Ghana OR Guinea OR Kenya OR Lesotho OR Liberia OR Madagascar OR Malawi OR Mali OR Mauritania OR Mauritius OR Mozambique OR Namibia OR Niger OR Nigeria OR Réunion OR Rwanda OR Sao Tome and Principe OR Senegal OR Seychelles OR Sierra Leone OR Somalia OR South Africa OR Sudan OR Swaziland OR Eswatini OR Tanzania OR Togo OR Uganda OR Western Sahara OR Zambia OR Zimbabwe) NOT (therapeutic* OR treatment* OR biomarker* OR HIV* OR **cervical cancer screening** OR **respiratory papillomatosis** OR **chlamydia trachomatis** OR **trichomonas vaginalis** OR **vaccine efficiency** OR **immunology** OR **cost-effectiveness** OR **microbiome** OR **surfactant protein** OR **self-sampling**))
Google scholar	allintitle: Africa HPV HPV OR HPV16 OR HPV18 OR HPV OR 6 OR HPV OR 5 OR HPV18 OR HPV OR 31 OR HPV OR 33 OR HPV OR 45 OR HPV52 OR coverage OR prevention OR uptake OR status OR access OR strategy OR guideline OR policy OR vaccine
Science direct	“HPV” AND (“vaccin” OR “cervix cancer prevention” OR “uptake” OR “hesitancy” OR “coverage”) AND (“Sub Saharan Africa”) NOT (“**therapeutic**” OR " **HIV** “)
African journals online	HPV AND (Gardasil OR Cevarix OR vaccin) AND (coverage OR uptake OR hesitancy OR acceptance OR barrier OR intervention OR strategy OR knowledge OR prevention OR access OR program OR guideline OR policy)

PRISMA guidelines were adopted based on the PICO acronym [35]. The PICO components of a research question were defined a priori by the study team and are as follow: patient/population, intervention, comparator, and outcomes [36]. In this literature search, the population refers to the general population of SSA, the intervention is defined as HPV-vaccination, the comparator are the different countries within SSA, and the outcome as the barriers and facilitators as outcome of this review. The following terms have been used to further refine the search: barriers, facilitators, hesitancy, willingness, acceptability, awareness, knowledge, behavior, and national programs.

Search strategies were adapted to each selected database using relevant search terms and combination of terms to a systematic search syntax with the Boolean “OR”, “AND” and “NOT” operators. Wildcards in form of asterixis were used to adapt for additional word endings [37]. Each syntax was adapted to the individual search requirements of the database. Additionally, the references of the selected articles were searched for similar relevant articles. However, the search didn’t yield for additional results.

### Eligibility criteria

Inclusion criteria: Publications listed within the past 10 years until 31 December 2021; studies concerning HPV vaccination programs, policies, and guidelines in SSA, which include all types of HPV vaccines; studies that investigated the coverage of HPV vaccination in SSA; studies that investigated hesitancy, willingness, acceptance, awareness, knowledge, and attitudes towards HPV vaccination in SSA; studies published in English, French, German, Italian, Spanish; studies listed in PubMed/MEDLINE, LIVIVO, Scopus, Science direct, AJOL, and/or Google Scholar database; target population of publication is in SSA.

Exclusion Criteria: Any article that does not have any of the above search keywords in its title or abstract; Unrelated, duplicated, unavailable full texts, or abstract-only papers; studies that focus exclusively e.g., on vaccine efficacy without being related to public health strategies, coverage, or access barriers; articles that focus only on CC screening; studies soley focused on HPV prevalence, HPV types causing recurrent respiratory papillomatosis, other genital infections (e.g., chlamydia trachomatis and trichomonas vaginalis), vaccine efficiency in terms of immunology, cost-effectiveness analysis, microbiome research, CC treatment, surfactant protein, and self-sampling.

### Data extraction strategy

The selected search results were exported from the databases and organized using Zotero citation manager software [38]. Duplicates were removed within the program. The remaining articles were transferred into the review management software Rayyan [39] ensuring a blinded voting process and systematic screening of the selected articles. Titles and abstracts were assessed against the inclusion and exclusion criteria by the three reviewers JK, PR and DF.

**Fig. 1:**
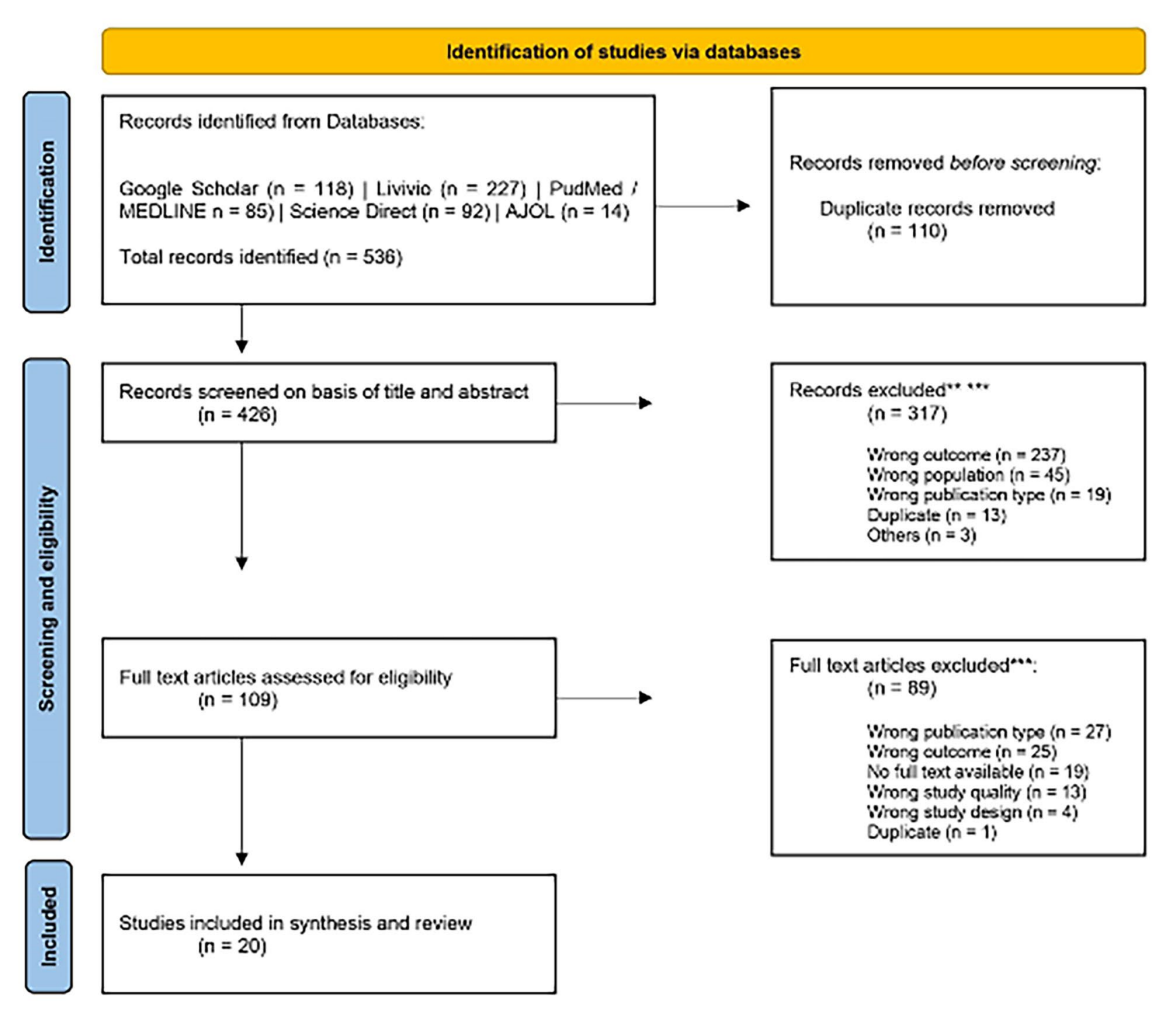
PRISMA Flowchart. From: Page MJ, McKenzie JE, Bossuyt PM, Boutron I, Hoffmann TC, Mulrow CD, et al. The PRISMA 2020 statement: an updated guideline for reporting systematic reviews. BMJ 2021;372:n71. doi: 10.1136/bmj.n71 | For more information, visit: http://www.prisma-statement.org/

After establishing fullfillment of inclusion and exclusion cirteria of the articles, full text articles were reviewed by JK and PR in a blinded double voting process. Conflicts were resolved through discussion until consens was reached after unblinding of the voting descisions in Rayyan. DF intervened in case of conflicts. The reasons for excluding full-text articles were recorded. The screening and exclusion process is based on the Moher model and is shown in the PRISMA flow diagram (Fig. 1) [40].

### Study characteristics

At first, abstracts were excluded from full-text evaluation with respect to the exclusion criteria.

The quality appraisal of the selected papers was then performed by JK and PR according to the JBI critical appraisal checklist to ensure methodological quality in systematic reviews [33]. Nine appraisal criteria were established: (i) address of target population, (ii) appropriateness of sampling, (iii) adequacy of sample size, (iv) appropriate description of subjects and settings, (v) sufficient coverage of identified sample, (vi) validity of methods, (vii) reliability of methods as well as (viii) appropriate response rates, and (ix) statistical analysis. All criteria were rated as “Y” if a criterion was met, “N” if not, “U” in case of uncertainties or lacking information and “N/A” if a criterion was not applicable for the reviewed study. Studies with five and more categories answered with “Y” were considered for inclusion. Ambiguities were discussed among PR and JK until consensus was reached. All articles passed the quality assessment as displayed in the supplementary Tables 1 and 2.

### Data synthesis and presentation

**Fig. 2:**
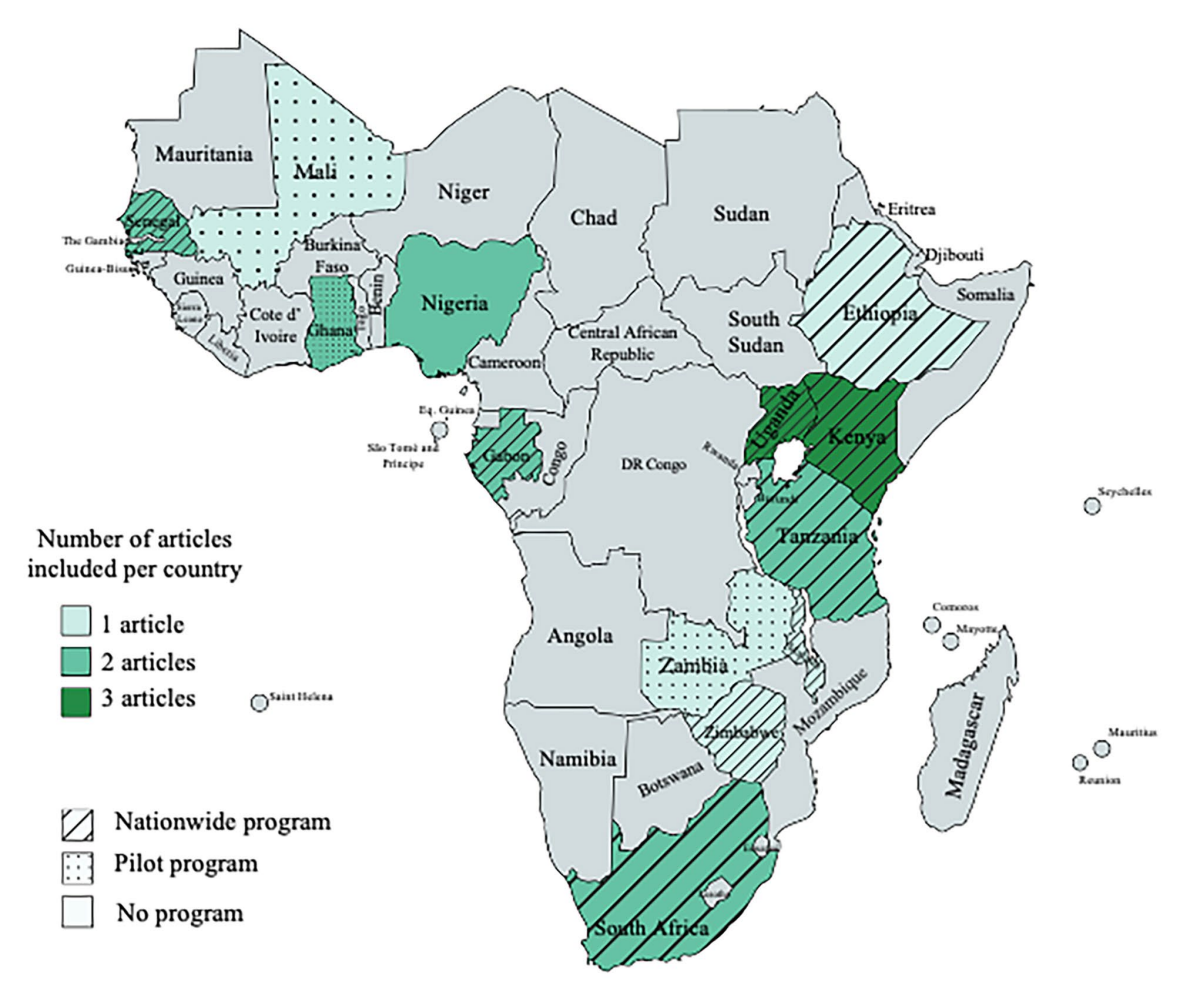
Countries where the studies of the selected articles took place. Different intensities of green depict the frequency of articles identified per country. Lines and dots describe the status of HPV vaccination programs in the countries (see legend), created with mapchart.net

**Fig. 3:**
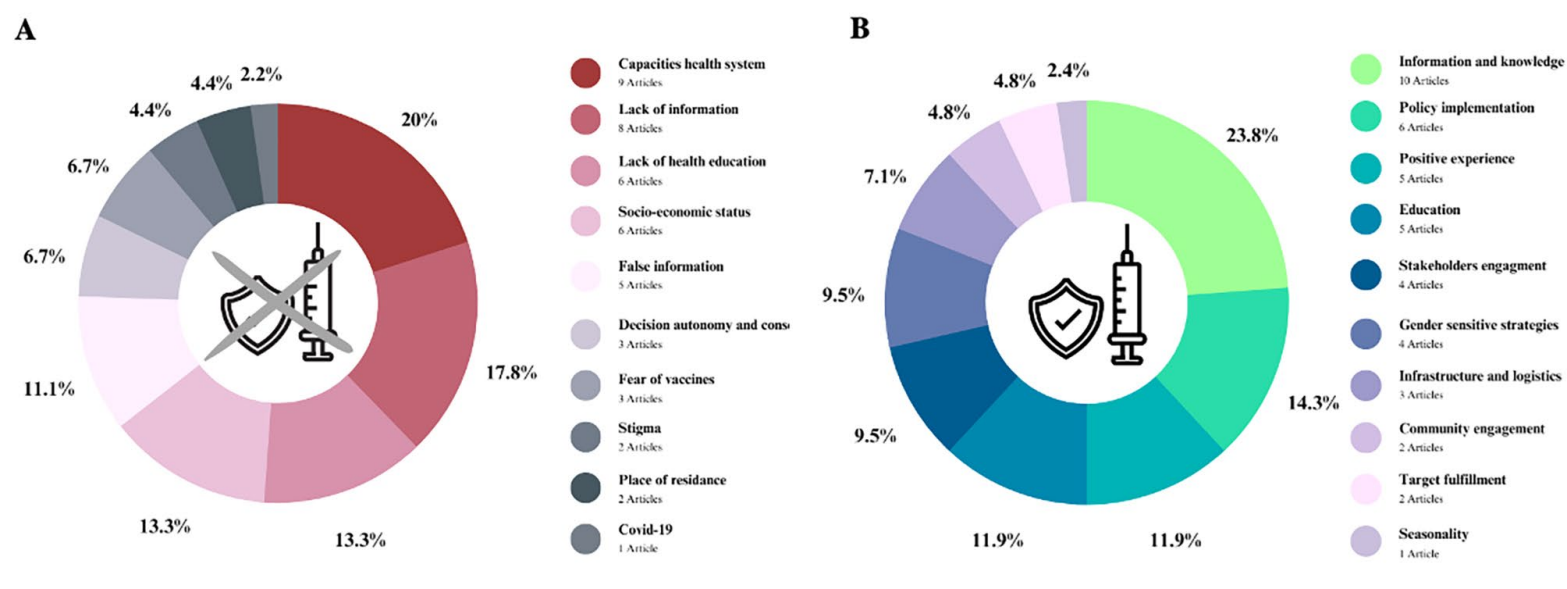
Barriers (**A**) and facilitators (**B**) for HPV vaccination in sub-Saharan Africa

Author(s) and year of publication, study design, country of research, instruments, target group, sample, sample characteristics, age of participants, outcome measures, and vaccination barriers and facilitators were extracted and tabulated for all selected articles according to the JBI guidelines [33]. All data were independently extracted by two researchers to ensure accuracy. Inconsistencies in the extracted data were resolved by the two researchers (JK and PR) until a consensus was reached. All authors cross-checked the table for errors and completeness. Strength and limitations of each study were evaluated on basis of the quality appraisal from the supplementary Tables 1 and 2. In addition to the PRISMA flowchart (Fig. 1) visualizing the identification of articles via databases, countries where the selected articles took place were displayed on a map (Fig. 2). Barriers and facilitators extracted from the tabulated tables were presented in a dedicated figure (Fig. 3 – A and B).

## Results

### Studies selection

A total of 536 records were retrieved through our systematic search from the selected databases. After the removal of 110 duplicates in Zotero, a total of 426 remaining article titles and abstracts were screened in Rayyan, removing an additional 317 articles. Most articles were removed if the study outcome or the study populations did not match with the criteria of this review. After full text assessment of 109 remaining articles, another 89 articles were excluded mostly because the publication type or study outcome did not match.

The total retrieval process excluded 516 articles and yielded 20 articles that were considered for the assessment of methodological quality. The selection process is displayed in detail in the PRISMA flowchart (Fig. 1).

Overall, the quality of studies selected was highly variable. For the quantitative studies, it ranged from five to nine points (Mean: 6.75 [SD = 1.01]) following the JBI critical appraisal approach.

All studies retrieved in the systematic literature search were selected for synthesis. The study by Garon et al. received the lowest score with a total of five points [41], while the highest score was assigned to the publication of Kassa et al. [42] which met all nine appraisal criteria. While all studies included appropriate targeting of sample population and sampling strategies, only eleven articles out of twenty provided sufficient information on the calculation of the sample size [42–52]. Sufficient statistical analysis was offered by all studies included.

The quality of the qualitative studies included in the review ranged from six to nine points (Mean: 7.25 [SD = 1.06]) following the JBI critical appraisal approach. All studies retrieved in the systematic literature search were selected for synthesis as they scored above five points. The lowest score of six points was assigned to the study by Massey et al. [50] and Delany-Morethwe et al. [51], and the highest score to three publications [48, 49, 52], which met all nine appraisal criteria. Frequently, the sample size and its selection were insufficiently or unclearly stated.

### Study characteristics

After selection, certain characteristics of the articles were extracted and summarized. Specifically, study design, study country, research instruments, target group and target population, sample characteristics, age of participants, outcome measures, barriers, and facilitators, are summarized in the supplementary Tables 3 and 4. After appraisal the content of the articles were organized into three main thematic areas: (i) barriers, (ii) facilitators, and (iii) HPV vaccination for boys in sub-Saharan Africa.

The studies described in the 20 articles selected were conducted in twelve different countries with a combined sample size of 10,396 participants. Age of the participants included ranged from 9 to 62 years. The mean age of the participants was reported in six articles, among these articles the overall mean was 32.32 (SD = 8.99).

The selected articles included three articles each (15%) from Uganda and Kenya, two articles (10%) from Nigeria, Tanzania, Ghana, South Africa, and Senegal, and one (5%) from Ethiopia, Zimbabwe, Mali, Malawi, and Zambia (Fig. 2).

Seven articles reported HPV vaccination coverage within their sample (median 17.6% [IQR = 21.7]) with a range from 5.6% vaccine coverage in children in Enebe et al. [43] to 66.5% coverage of primary school female students in Kassa et al. [42]. Willingness to vaccinate against HPV was reported in five articles included in the literature review (Median 74.5% [IQR = 34.2]) with a range of 28.0% of adolescents in Massey et al. [50] to 88.1% of girls aged eight to eighteen in Vermandere et al. [53].

### Barriers to HPV vaccination in sub-saharan Africa

The barrier for successful HPV vaccination most frequently identified (Fig. 3A) among nine articles [7, 41, 43, 45, 48, 49, 51, 54, 55] related to the scarcity of resources, such as the lack of availability of materials to raise awareness [41], inadequate cold chain capacities [51], lack of personnel [51], and limited capacities for the management of possible adverse events [51].

Lack of information regarding the vaccination services was highlighted in eight articles [41, 45, 47, 50, 53, 55–57] as a barrier to HPV vaccination. For example, the target groups of the vaccination campaigns did not have sufficient information on how to reach the vaccination sites. This factor was mostly identified within the general population as the recipient/user of this type of health care service [53].

Further, limited knowledge of HPV and its pathogenesis was identified as a barrier in six articles [45, 47, 48, 50, 51, 53]. For instance, a lack of HPV awareness cannot only affect individuals’ understanding of the need for vaccination [47, 48, 50] but limited HPV health literacy among e.g., school teachers can also affect the quality of HPV vaccination information sessions [58], and among parents to consent to the vaccination of their children [51].

Moreover, misinformation about, for example, vaccine side effects [41] and safety of the vaccines [52] was another obstacle to vaccination described in five articles [41, 48, 51, 56, 57]. Milondzo et al. identified misinformation as the main driver of negative attitudes towards HPV vaccination, which resulted in low vaccination rates during their study [57]. Additionally, parents of unvaccinated girls were more likely to be influenced by online articles, like vaccine injury reports, than parents of vaccinated girls [57]. Further, in three articles [7, 41, 58] it was reported that the belief that HPV vaccination could affect girls’ fertility represented a strong barrier to vaccination. Finally, Poole et al., reported that in Mali there was the belief that men could not contract the virus and that the population was scarcely aware that HPV is an STI [59].

Two studies found that the stigma around sexually transmitted infections, such as HPV can prevent individuals from getting vaccinated [55, 58].

One article specifically explored acceptability of HPV vaccination among health care workers (HCW) [45]. Despite the participants’ overall high levels of knowledge of CC, its risk factors and the role of HPV infection, only 17.6% of the respondents, eligible for the vaccination, had received at least one dose of the HPV vaccine [45].

Other relevant but less reported barriers include: limited access to vaccines in rural areas [42, 50], and the fear of pain related to vaccine injections [41]. Interestingly, as HPV vaccination campaigns are commonly implemented in schools, COVID-19 represented a barrier since temporary school closures led to the discontinuation of vaccination campaigns [42, 44, 45, 47, 48, 50, 57, 59, 60].

### Facilitators for HPV vaccination in sub-saharan Africa

Firstly, correct knowledge of HPV, the HPV vaccine, and the consequences of CC were cited as the most important facilitators of both HPV vaccination uptake and willingness to get vaccinated (Fig. 3B) in ten articles [41, 42, 44, 45, 47, 48, 50, 57, 59, 60]. Being knowledgeable about the HPV vaccine was associated with vaccine uptake in Ethiopia as described in Kassa et al. [42].

Secondly, six articles reported that the integration of HPV vaccination into routine vaccination campaigns can facilitate HPV vaccination uptake [41, 42, 50, 55, 56, 61]. The engagement of policy makers at the community as well as at the national level were described as key factors in promoting HPV vaccination in four articles [57, 41, 50, 58].

Further, empowering women by assigning them the role of peer community leaders is described as a strong facilitator by four articles [46, 58–60]. Interestingly, according to Asare et al. women who take on the role of community peer leaders are more likely to get the first vaccination dose and to complete the recommended number of doses [46].

Five studies [43, 48, 49, 52, 60] identified the positive experience with a previous other non HPV related vaccination as a facilitator to get vaccinated against HPV. Specifically, previous history of vaccination of respondents was associated with the acceptance of HPV vaccination of their children and/or relatives [43].

Less frequently cited facilitators in the appraised articles included high parental educational attainment [44, 47, 50, 54, 57], ease of access to vaccination site as described in three articles [47, 50, 51], and one article highlighted that vaccination campaigns conducted during dry seasons are preferred [51]. Clear vaccination strategies for HCWs and local stakeholders have also been identified as a facilitator of vaccination uptake [41, 49]. For instance, standardized criteria can help HCWs, as well as teachers and community leaders, to identify who is eligible for vaccinations. A study in Zimbabwe shows that, having different criteria for vaccination of school-aged girls (by grade) and non-school-aged girls (by age) can lead to confusion [41], and opted for a campaign that uniformly vaccinated all girls in fifth grade according to grade level [41].

### HPV vaccination of boys in sub-saharan Africa

Muhwezi et al. was the only article yielded by this review, which reported on HPV and possible HPV vaccination for boys [44]. The cross-sectional survey was conducted in Uganda and assessed parent’s knowledge, risk perception and willingness to allow their son(s) (secondary school boys aged 10–23 years) to get vaccinated against HPV in the future. Findings show that parents were less willing to have their sons vaccinated (78.3% willingness to vaccinate) than their daughters (90.6% willingness to vaccinate). Within the sample, 36% of parents thought that their sons might be at risk of contracting HPV. Parents not willing to have their sons vaccinated were more likely to believe that their sons could not contract HPV (Crude OR: 2.72, 95% CI: 1.24–5.95, x2 = 6.63, p = 0.01 ), and did not know that HPV is transmissible through sexual contact (Crude OR: 2.23, 95% CI: 1.31–3.80, x2 = 9.17, p = 0.002) [44].

## Discussion

HPV vaccination is recognized as the most effective measure to prevent CC as well as other types of HPV-driven cancers affecting both women and men [62]. Since the market entry of vaccines against HPV in 2006 [63], several countries have adopted different HPV vaccination strategies aimed at controlling HPV infections as well as onset and progression of different types of cancer [5]. While high-income and few middle-income countries have made enormous progress in the implementation of HPV-vaccination strategies [64] with 80% of countries adopting the vaccine in less than a decade, low-income countries, in particular those of SSA, lag far behind in terms of roll-out and coverage [65]. This is in part because of the later start date (with a peak registered in 2019) and implementation barriers [12, 17], but also simply because there are more LMICs than there are high income countries. At the same time, the WHO has highlighted that the current global HPV vaccination shortage, foreseen to last at least until 2024, might delay the introduction of HPV vaccination in those countries that are most in need [66]. These factors combined with the implementation challenges that became evident during the global COVID-19 vaccination campaign especially in SSA [56], risk to delay the WHO 90/70/90 strategy to eliminate CC. While the standard recommendation is currently a two doses vaccination schedule, a one dose schedule is under consideration to address the problem even though data to support the adaptation of the strategy is still insufficient [66–68].

Our systematic review has identified important barriers and facilitators for the successful implementation of HPV vaccination programs in SSA. Additionally, it shows that boys are scarcely considered for vaccination, highlighting the gender gap, which exists for HPV vaccination despite strong evidence for the relevance of this target group and the provision of the vaccine also to boys in many high-income countries [17]. To the best of our knowledge, this study represents the first systematic review investigating factors influencing HPV vaccination coverage in SSA. While the countries of SSA are diverse in terms of their infrastructures and socio-anthropological characteristics, they share certain programmatic strategies that have proven to be successful in the last decades, especially in terms of vaccination. We believe that the identification of common barriers and facilitators can promote joint actions at sub-regional level to drive and influence policies for a better and more successful coverage of HPV vaccines in the next decade in SSA. These findings will be crucial to contribute to the WHO 90/70/90 strategy to eliminate CC since the majority of girls under 15 years of age live in SSA [69] and to support the 12 SSA countries that are projected to introduce the vaccination in 2023 according to PATH [70].

After our search, a total of 20 articles were appraised and among those, more barriers than facilitators were identified in SSA where HPV vaccination programs have been implemented through national roll out strategies or pilot programs [32, 71](Fig. 2).

While misinformation and lack of health literacy are most commonly recognized as common barriers towards vaccine hesitancy in many populations [72, 73] and for different types of vaccines [74], our search identified the scarcity of resources as a key obstacle to the successful implementation of HPV vaccination programs in SSA. Nine out of 20 articles [7, 41, 43, 45, 48, 49, 51, 54, 55] report that the lack of resources represents the main barrier for the successful roll-out of HPV vaccination in nine countries in SSA. The high workload [48] alongside the limited capacities to provide high quality services [41] have been identified as important barriers to HPV vaccine provision and uptake. This stands in contrast to the experience of implementing routine child vaccinations, where many SSA countries have had tremendous successes in terms of increasing access to immunization and reducing child deaths [75]. The lack of capacities for the roll-out of vaccines in SSA has become evident once more during the COVID-19 pandemic [76]. Once the bottleneck of dose availability was unblocked because of increased production and the decreasing demand of high-income countries, many SSA countries were not in the position to cope with the in-country distribution costs to achieve the required vaccine coverage [77, 78]. In addition, the lower COVID-19 burden in many SSA countries compared to the rest of the world [79] delayed the political commitment to engage and allocate resources to primary prevention probably due to the perceived lack of medical need [80–82]. Similarly, we can speculate that CC and cancer in general still represent an unmet medical need in SSA [83]. This stands in contrast to the call of the SDG3 and the 2017 cancer resolution (WHA70.12), which stipulate that reducing the global cancer burden is a prerequisite for addressing social and economic inequity, stimulating economic growth, and accelerating sustainable development [84]. According to the WHO an estimated 70% of cancer deaths occur in LMICs and by 2030 LMICs are expected to bear the brunt of the projected 24.1 million new cancer cases per year. However, currently only eight of the 49 SSA countries have adopted nationwide HPV vaccination programs and merely three of the 49 [85] have implemented more than one pilot roll-out, qualifying for additional support resources e.g. through GAVI.

Health literacy and misinformation are among the leading factors influencing vaccine willingness for many types of vaccines in different parts of the world [86]. The WHO had already named vaccine hesitancy as one of the top ten global health threats in 2019 [87] and the COVID-19 pandemic has further shown its potential in hampering vaccination uptake. Unprecedent attention has been given to vaccine hesitancy thanks to the COVID-19 pandemic showing that misconceptions, misinformation and uncontrolled information on vaccines have a global detrimental effect regardless of the region of the world [88]. Misconceptions, such as vaccines being a source of infertility, have been identified as a barrier for HPV vaccination in a study conducted in Kenya. Notably, individuals interviewed in the selected studies were unaware that HPV is an STI [55, 59] and believed that men cannot contract the virus [59]. Interestingly, similar findings occurred in high-income countries [89] where gender neutral vaccination programs are being promoted and implemented. The social stigma and the gender polarization implied in these findings show the importance of increased education, awareness raising and community engagement among both the HCWs and the general population when a country engages in the adoption of HPV vaccination. The HIV pandemic has clearly shown that social stigma produces a strong detrimental effect against any medical measures if these can disclose or implicate any sensitive behavior [90]. Notably, among the 20 articles selected for this systematic review, only one assessed parents’ attitude in supporting HPV vaccination for boys. The study conducted in Uganda showed that parents were less likely to be willing to have their sons vaccinated (78.3% willingness to vaccinate) as compared to their daughters (90.6% willingness to vaccinate), and most of the parents believed that boys have an overall lower risk of contracting the infection [44]. Even though the current guidelines of the WHO for low- and middle-income countries encourage HPV vaccinations for young and adolescent girls [68] many high-income countries have adopted HPV vaccinations for all individuals regardless of their gender [91, 92]. This strategy not only contributes to a higher effectiveness of the vaccine, but it also reduces the stigma around the HPV infection. However, the awareness of HPV infection in males and the fact that they can also benefit from HPV vaccination, is still low also in those countries where vaccination strategies are also targeting boys. This indicates that overall a gender-related barrier against HPV vaccination prevails [89].

Moreover, the COVID-19 pandemic has highlighted additional structural barriers associated with HPV vaccination programs in SSA [56]. In this region of the world, HPV vaccination is frequently implemented in schools [93]. This meant that school closures during the COVID-19 pandemic interrupted HPV vaccination services in many of these countries [94]. Vaccination programs are generally among the first services to be interrupted or discontinued in emergencies, such as natural disasters, civil conflicts or wars [95]. Thus, establishing national and sub-regional plans for structured and resilient HPV vaccination programs is crucial, not only in order to avoid repeated discontinuation of services but also to increase population trust in the health services offered.

In addition, a number of facilitators were identified as part of this review, which can inform HPV vaccination guidelines/strategies and interventions for countries south of the Sahara. As is the case for many other vaccination programs, knowledge and ease of access were the main factors facilitating HPV vaccination. In particular, the role of peer leaders was highlighted as an important driver of vaccine uptake [60]. Community engagement has proven to be a powerful tool to ensure the success of health interventions in both high and low- and middle-income countries. Indeed, some of the dynamics of health intervention strategies are becoming a global phenomenon independently from differences in resources and culture [96]. Finally, positive experiences with vaccinations in the past encourage HPV vaccination [48, 52, 54, 60]. Since in the aftermath of the COVID-19 pandemic, the infodemic around COVID-19 vaccination [97] as well as misconceptions [98], and political manipulation [99] has created discomfort with the concept of vaccination in general, this should be promptly addressed through tailored health communication strategies since it could clearly affect the success of other vaccinations, including HPV.

Finally, while this study synthesizes common barriers and facilitators of HPV vaccination strategies in SSA, including the role of stigma, it is not without limitations. Firstly, the different methodologies applied in the selected articles, did not allow for frequency weighting, or further analysis such as meta-analysis. A main limitation of this systematic review is that the articles selected covered only 12 out of the 49 countries belonging to the region of SSA, moreover policies and reports (i.e., from international NGOs) were not systematically retrieved through our search syntax across the different databases. Finally, our review only considers articles written in English, German, Italian, French or Spanish. Articles in other languages than these were not considered for this review, all articles included in this review are in English, therefore a language bias cannot be excluded.

### Conclusions

**Fig. 4:**
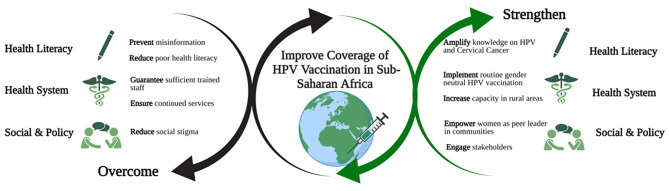
Factors to improve coverage of HPV Vaccination in Sub-Saharan Africa, created with BioRender.com

In conclusion, this systematic review synthesizes valuable information on elements that can inhibit or facilitate the successful implementation of HPV vaccination programs in SSA. As summarized in Fig. 4, we recommend actions to prevent misinformation and social stigma, invest in health literacy for both health services users and providers, and limit shortages in supply chains at every level. Moreover, we recommend to invest in decentralized solutions and in the engagement of peer-leaders to increase accessibility and coverage. Lessons learnt from past experiences of countries in this region, can contribute to the design and implementation of more effective national HPV vaccination programs based on good practices to meet the target of the WHO 90/70/90 triple intervention strategy to eliminate CC.

## Electronic supplementary material

Below is the link to the electronic supplementary material.

Supplementary Material 1

Supplementary Material 2

Supplementary Material 3

Supplementary Material 4

## Data Availability

Additional datasets used during the current study are available from the corresponding author on reasonable request. All relevant data, such as the search syntax used, is already visible in the review.

## Abbreviations

AJOL: African Journals Online
CC: Cervical Cancer
CDC: The American Center of Disease Control
HPV: Human Papilloma Virus
HR-HPV: High-risk HPV types
JBI: The Joanna Briggs Institute
LMICs: Low and Middle-Income Countries
MSM: Men who have Sex with Men
NGO: Non-Governmental Organizations
PATH: Program for Appropriate Technology in Health
PICO: Patient/population, Intervention, Comparator, and Outcomes
PRISMA: Preferred Reporting Items for Systematic Reviews and Meta-Analyses
PROSPERO: International Prospective Register of Systematic Reviews
SSA: Sub-Saharan Africa
WHO: World Health Organization

## References

[1] Gheit T. Mucosal and Cutaneous Human Papillomavirus Infections and Cancer Biology. Front Oncol. 2019;9. 10.3389/fonc.2019.00355 PMC6517478 31134154

[2] Hathaway JK (2012). HPV: diagnosis, Prevention, and treatment. Clin Obstet Gynecol.

[3] de Villiers E-M, Fauquet C, Broker TR, Bernard H-U, zur Hausen H (2004). Classification of papillomaviruses. Virology.

[4] Burd EM (2003). Human papillomavirus and cervical cancer. Clin Microbiol Rev.

[5] de Martel C, Plummer M, Vignat J, Franceschi S (2017). Worldwide burden of cancer attributable to HPV by site, country and HPV type. Int J Cancer.

[6] Brianti P, Flammineis ED, Mercuri SR. Review of HPV-related diseases and cancers. 2017;:6. 28368072

[7] Liu Z, Rashid T, Nyitray AG (2016). Penises not required: a systematic review of the potential for human papillomavirus horizontal transmission that is non-sexual or does not include penile penetration. Sex Health.

[8] Handler MZ, Handler NS, Majewski S, Schwartz RA (2015). Human papillomavirus vaccine trials and tribulations: clinical perspectives. J Am Acad Dermatol.

[9] De Vuyst H, Alemany L, Lacey C, Chibwesha CJ, Sahasrabuddhe V, Banura C (2013). The Burden of Human Papillomavirus Infections and related Diseases in Sub-Saharan Africa. Vaccine.

[10] Markowitz LE, Tsu V, Deeks SL, Cubie H, Wang SA, Vicari AS (2012). Human papillomavirus vaccine introduction–the first five years. Vaccine.

[11] WHO. Human papillomavirus vaccines (HPV). 2023. https://www.who.int/teams/immunization-vaccines-and-biologicals/diseases/human-papillomavirus-vaccines-(HPV). Accessed 2 Apr 2023.

[12] Gavi, The Vaccine Alliance. Human papillomavirus vaccine support. 2022. https://www.gavi.org/types-support/vaccine-support/human-papillomavirus. Accessed 14 Dec 2022.

[13] Falcaro M, Castañon A, Ndlela B, Checchi M, Soldan K, Lopez-Bernal J (2021). The effects of the national HPV vaccination programme in England, UK, on cervical cancer and grade 3 cervical intraepithelial neoplasia incidence: a register-based observational study. The Lancet.

[14] Hildesheim A, Wacholder S, Catteau G, Struyf F, Dubin G, Herrero R (2014). Efficacy of the HPV-16/18 vaccine: final according to protocol results from the blinded phase of the randomized Costa Rica HPV-16/18 vaccine trial. Vaccine.

[15] Garland SM, Hernandez-Avila M, Wheeler CM, Perez G, Harper DM, Leodolter S (2007). Quadrivalent vaccine against human papillomavirus to prevent anogenital diseases. N Engl J Med.

[16] Porras C, Tsang SH, Herrero R, Guillén D, Darragh TM, Stoler MH (2020). Efficacy of the bivalent HPV vaccine against HPV 16/18-associated precancer: long-term follow-up results from the Costa Rica Vaccine Trial. Lancet Oncol.

[17] Bruni L, Saura-Lázaro A, Montoliu A, Brotons M, Alemany L, Diallo MS (2021). HPV vaccination introduction worldwide and WHO and UNICEF estimates of national HPV immunization coverage 2010–2019. Prev Med.

[18] Díez-Domingo J, Sánchez-Alonso V, Villanueva R-J, Acedo L, Tuells J (2021). Impact of a gender-neutral HPV vaccination program in men who have sex with men (MSM). Int J Environ Res Public Health.

[19] Fairley CK, Zou H, Zhang L, Chow EPF (2017). Human papillomavirus vaccination in men who have sex with men - what will be required by 2020 for the same dramatic changes seen in heterosexuals. Sex Health.

[20] Man I, Georges D, de Carvalho TM, Saraswati LR, Bhandari P, Kataria I et al. Evidence-based impact projections of single-dose human papillomavirus vaccination in India: a modelling study. Lancet Oncol. 2022;0. 10.1016/S1470-2045(22)00543-5 PMC9622421 36174583

[21] Brisson M, Kim JJ, Canfell K, Drolet M, Gingras G, Burger EA (2020). Impact of HPV vaccination and cervical screening on cervical cancer elimination: a comparative modelling analysis in 78 low-income and lower-middle-income countries. Lancet Lond Engl.

[22] Ferlay et al. J. Global Cancer Observatory: Cancer Today. Lyon, France: International Agency for Research on Cancer. 2020. http://gco.iarc.fr/today/home. Accessed 31 Oct 2022.

[23] Hull R, Mbele M, Makhafola T, Hicks C, Wang S-M, Reis RM (2020). Cervical cancer in low and middle-income countries. Oncol Lett.

[24] Sung H, Ferlay J, Siegel RL, Laversanne M, Soerjomataram I, Jemal A (2021). Global Cancer Statistics 2020: GLOBOCAN estimates of incidence and Mortality Worldwide for 36 cancers in 185 countries. CA Cancer J Clin.

[25] Aggarwal P (2014). Cervical cancer: can it be prevented?. World J Clin Oncol.

[26] Black E, Richmond R (2018). Prevention of Cervical Cancer in Sub-Saharan Africa: the Advantages and Challenges of HPV Vaccination. Vaccines.

[27] WHO (2020). Global strategy to accelerate the elimination of cervical cancer as a public health problem.

[28] Shinkafi-Bagudu Z. Global Partnerships for HPV Vaccine Must Look Beyond National Income. JCO Glob Oncol. 2020;6:GO.20.00504. 10.1200/GO.20.00504 PMC7713535 33186079

[29] Denny L (2022). HPV vaccine introduction and implementation in low- and Middle-Income Countries. Vaccine.

[30] Eric Asempah. Cervical Cancer Prevalence in sub-Saharan Africa and HPV Vaccination Policy: A Public Health Grand Challenge? J Cancer Immunol. 2021;3.

[31] Ngcobo N, Jaca A, Iwu-Jaja CJ, Mavundza E (2021). Reflection: burden of cervical cancer in Sub-Saharan Africa and progress with HPV vaccination. Curr Opin Immunol.

[32] Tsu VD, LaMontagne DS, Atuhebwe P, Bloem PN, Ndiaye C (2021). National implementation of HPV vaccination programs in low-resource countries: Lessons, challenges, and future prospects. Prev Med.

[33] The Joanna Briggs Institute. The Joanna Briggs Institute Reviewers’ Manual. 2015.

[34] Page MJ, McKenzie JE, Bossuyt PM, Boutron I, Hoffmann TC, Mulrow CD et al. The PRISMA 2020 statement: an updated guideline for reporting systematic reviews. BMJ. 2021;:n71. 10.1136/bmj.n71 PMC8005924 33782057

[35] Oxman AD, Sackett DL, Guyatt GH (1993). Users’ guides to the medical literature. I. How to get started. The evidence-based Medicine Working Group. JAMA.

[36] Brockmeier AJ, Ju M, Przybyła P, Ananiadou S (2019). Improving reference prioritisation with PICO recognition. BMC Med Inform Decis Mak.

[37] Toby Port. PubMed: Basic Boolean Search Hints. NLM Technical Bulletin. Jul-Aug 1997. 1997. https://www.nlm.nih.gov/pubs/techbull/ja97/ja97_pubmed.html. Accessed 31 Oct 2022.

[38] Corporation for Digital Scholarship. Zotero. 2006.

[39] Ouzzani M, Hammady H, Fedorowicz Z, Elmagarmid A (2016). Rayyan—a web and mobile app for systematic reviews. Syst Rev.

[40] Moher D, Liberati A, Tetzlaff J, Altman DG, for the PRISMA Group (2009). Preferred reporting items for systematic reviews and meta-analyses: the PRISMA statement. BMJ.

[41] Garon JR, Mukavhi A, Rupfutse M, Bright S, Brennan T, Manangazira P (2022). Multiple cohort HPV vaccination in Zimbabwe: 2018–2019 program feasibility, awareness, and acceptability among health, education, and community stakeholders. Vaccine.

[42] Kassa HN, Bilchut AH, Mekuria AD, Lewetie EM (2021). Practice and Associated factors of human papillomavirus vaccination among Primary School students in Minjar-Shenkora District, North Shoa Zone, Amhara Regional State, Ethiopia, 2020. Cancer Manag Res.

[43] Enebe JT, Enebe NO, Agunwa CC, Nduagubam OC, Okafor II, Aniwada EC et al. The awareness, acceptability and uptake of cervical cancer vaccination services among female secondary school teachers in Enugu, Nigeria: a cross-sectional study. Pan Afr Med J. 2021;39. 10.11604/pamj.2021.39.62.28824 PMC8363950 34422185

[44] Muhwezi WW, Banura C, Turiho AK, Mirembe F (2014). Parents’ knowledge, risk perception and willingness to allow young males to receive human papillomavirus (HPV) vaccines in Uganda. PLoS ONE.

[45] Ebu NI, Abotsi-Foli GE, Gakpo DF (2021). Nurses’ and midwives’ knowledge, attitudes, and acceptance regarding human papillomavirus vaccination in Ghana: a cross-sectional study. BMC Nurs.

[46] Asare M, Agyei-Baffour P, Lanning BA, Barimah Owusu A, Commeh ME, Boozer K (2020). Multi-Theory Model and Predictors of Likelihood of accepting the Series of HPV Vaccination: a cross-sectional study among ghanaian adolescents. Int J Environ Res Public Health.

[47] Ezenwa B, Balogun, Okafor. Mothers’ human papilloma virus knowledge and willingness to vaccinate their adolescent daughters in Lagos, Nigeria. Int J Womens Health. 2013;:371. 10.2147/IJWH.S44483 PMC3711756 23874123

[48] Remes P, Selestine V, Changalucha J, Ross DA, Wight D, de Sanjosé S (2012). A qualitative study of HPV vaccine acceptability among health workers, teachers, parents, female pupils, and religious leaders in northwest Tanzania. Vaccine.

[49] Nabirye J, Okwi LA, Nuwematsiko R, Kiwanuka G, Muneza F, Kamya C (2020). Health system factors influencing uptake of human papilloma virus (HPV) vaccine among adolescent girls 9–15 years in Mbale District, Uganda. BMC Public Health.

[50] Massey PM, Boansi RK, Gipson JD, Adams RM, Riess H, Dieng T (2017). Human papillomavirus (HPV) awareness and vaccine receptivity among senegalese adolescents. Trop Med Int Health.

[51] Delany-Moretlwe S, Kelley KF, James S, Scorgie F, Subedar H, Dlamini NR (2018). Human papillomavirus vaccine introduction in South Africa: implementation Lessons from an evaluation of the National School-Based vaccination campaign. Glob Health Sci Pract.

[52] Turiho AK, Okello ES, Muhwezi WW, Katahoire AR (2017). Perceptions of human papillomavirus vaccination of adolescent schoolgirls in western Uganda and their implications for acceptability of HPV vaccination: a qualitative study. BMC Res Notes.

[53] Vermandere H, Naanyu V, Mabeya H, Vanden Broeck D, Michielsen K, Degomme O (2014). Determinants of Acceptance and subsequent uptake of the HPV Vaccine in a cohort in Eldoret, Kenya. PLoS ONE.

[54] Kisaakye E, Namakula J, Kihembo C, Kisakye A, Nsubuga P, Babirye JN. Level and factors associated with uptake of Human papillomavirus infection vaccine among female adolescents in Lira District, Uganda. Pan Afr Med J. 2018;31. 10.11604/pamj.2018.31.184.14801 PMC6488240 31086634

[55] Mabeya H, Odunga J, Broeck DV. Mothers of adolescent girls and Human Papilloma Virus (HPV) vaccination in Western Kenya. Pan Afr Med J. 2021;38. 10.11604/pamj.2021.38.126.21359 PMC8051220 33912296

[56] Li AJ, Manzi F, Kyesi F, Makame Y, Mwengee W, Fleming M et al. Tanzania’s human papillomavirus (HPV) vaccination program: Community awareness, feasibility, and acceptability of a national HPV vaccination program, 2019. Vaccine. 2022;40:A38–48. 10.1016/j.vaccine.2021.06.047 PMC9601816 34229889

[57] Milondzo T, Meyer JC, Dochez C, Burnett RJ (2021). Misinformation drives low human papillomavirus Vaccination Coverage in South African Girls attending private schools. Front Public Health.

[58] Vermandere H, Naanyu V, Degomme O, Michielsen K (2015). Implementation of an HPV vaccination program in Eldoret, Kenya: results from a qualitative assessment by key stakeholders. BMC Public Health.

[59] Poole DN, Tracy JK, Levitz L, Rochas M, Sangare K, Yekta S (2013). A cross-sectional study to assess HPV Knowledge and HPV Vaccine Acceptability in Mali. PLoS ONE.

[60] Ports KA, Reddy DM, Rameshbabu A (2013). Barriers and facilitators to HPV Vaccination: perspectives from Malawian Women. Women Health.

[61] Njuguna DW, Mahrouseh N, Isowamwen OV, Varga O, Knowledge (2021). Attitude and practice of Main Stakeholders towards human papilloma virus infection and vaccination in Mombasa and Tana-River Counties in Kenya: a qualitative study. Vaccines.

[62] Harper DM, Franco EL, Wheeler CM, Moscicki A-B, Romanowski B, Roteli-Martins CM (2006). Sustained efficacy up to 4.5 years of a bivalent L1 virus-like particle vaccine against human papillomavirus types 16 and 18: follow-up from a randomised control trial. Lancet Lond Engl.

[63] Human papillomavirus vaccines (2017). WHO position paper, May 2017. Releve Epidemiol Hebd.

[64] Spayne J, Hesketh T (2021). Estimate of global human papillomavirus vaccination coverage: analysis of country-level indicators. BMJ Open.

[65] Dorji T, Nopsopon T, Tamang ST, Pongpirul K (2021). Human papillomavirus vaccination uptake in low-and middle-income countries: a meta-analysis. EClinicalMedicine.

[66] Colzani E, Johansen K, Johnson H, Pastore Celentano L. Human papillomavirus vaccination in the European Union/European Economic Area and globally: a moral dilemma. Eurosurveillance. 2021;26. 10.2807/1560-7917.ES.2021.26.50.2001659 PMC8728487 34915976

[67] Lehtinen M, Pimenoff VN. Moral dilemma(s) in human papillomavirus vaccination – revisiting the role of the herd effect. Eurosurveillance. 2021;26. 10.2807/1560-7917.ES.2021.26.50.2101154 PMC8728494 34915973

[68] One-dose Human. Papillomavirus (HPV) vaccine offers solid protection against cervical cancer. https://www.who.int/news/item/11-04-2022-one-dose-human-papillomavirus-(hpv)-vaccine-offers-solid-protection-against-cervical-cancer. Accessed 25 Nov 2022. PMC9280593 35537734

[69] Population. ages 15–64, female (% of female population) | Data. https://data.worldbank.org/indicator/SP.POP.1564.FE.ZS?view=map. Accessed 2 Apr 2023.

[70] Path. Global HPV, Vaccine Introduction. Overview. 2023. https://www.path.org/resources/global-hpv-vaccine-introduction-overview/. Accessed 18 Apr 2023.

[71] Gallagher KE, Howard N, Kabakama S, Mounier-Jack S, Griffiths UK, Feletto M (2017). Lessons learnt from human papillomavirus (HPV) vaccination in 45 low- and middle-income countries. PLoS ONE.

[72] Lorini C, Santomauro F, Donzellini M, Capecchi L, Bechini A, Boccalini S (2018). Health literacy and vaccination: a systematic review. Hum Vaccines Immunother.

[73] Zhang H, Li Y, Peng S, Jiang Y, Jin H, Zhang F (2022). The effect of health literacy on COVID-19 vaccine hesitancy among community population in China: the moderating role of stress. Vaccine.

[74] Johri M, Subramanian SV, Sylvestre M-P, Dudeja S, Chandra D, Koné GK (2015). Association between maternal health literacy and child vaccination in India: a cross-sectional study. J Epidemiol Community Health.

[75] Adamu AA, Jalo RI, Habonimana D, Wiysonge CS. Since January 2020 Elsevier has created a COVID-19 resource centre with free information in English and Mandarin on the novel coronavirus COVID- 19. The COVID-19 resource centre is hosted on Elsevier Connect, the company ’ s public news and information. 2020;January.

[76] Masresha B, Ruiz MAS, Atuhebwe P, Mihigo R. The first year of COVID-19 vaccine roll-out in Africa: challenges and lessons learned. Pan Afr Med J. 2022;41. 10.11604/pamj.supp.2022.41.2.33686 PMC9474932 36159028

[77] Frontiers | COVID-19 Vaccination in Lower-Middle Income Countries. : National Stakeholder Views on Challenges, Barriers, and Potential Solutions. https://www.frontiersin.org/articles/10.3389/fpubh.2021.709127/full. Accessed 25 Nov 2022. 10.3389/fpubh.2021.709127 PMC8377669 34422750

[78] Mobarak AM, Miguel E, Abaluck J, Ahuja A, Alsan M, Banerjee A (2022). End COVID-19 in low- and middle-income countries. Science.

[79] Ackah BBB, Woo M, Stallwood L, Fazal ZA, Okpani A, Ukah UV (2022). COVID-19 vaccine hesitancy in Africa: a scoping review. Glob Health Res Policy.

[80] Alam ST, Ahmed S, Ali SM, Sarker S, Kabir G, Ul-Islam A (2021). Challenges to COVID-19 vaccine supply chain: implications for sustainable development goals. Int J Prod Econ.

[81] Burgess RA, Osborne RH, Yongabi KA, Greenhalgh T, Gurdasani D, Kang G (2021). The COVID-19 vaccines rush: participatory community engagement matters more than ever. Lancet Lond Engl.

[82] Lancet Commission on COVID-19 Vaccines and Therapeutics Task Force Members (2021). Urgent needs of low-income and middle-income countries for COVID-19 vaccines and therapeutics. Lancet Lond Engl.

[83] Bray F, Ferlay J, Soerjomataram I, Siegel RL, Torre LA, Jemal A (2018). Global cancer statistics 2018: GLOBOCAN estimates of incidence and mortality worldwide for 36 cancers in 185 countries. CA Cancer J Clin.

[84] Seventieth World Health Assembly. Cancer prevention and control in the context of an integrated approach. 2017.

[85] Amponsah-Dacosta E, Blose N, Nkwinika VV, Chepkurui V. Human Papillomavirus Vaccination in South Africa: Programmatic Challenges and Opportunities for Integration With Other Adolescent Health Services? Front Public Health. 2022;10. 10.3389/fpubh.2022.799984 PMC8841655 35174123

[86] Galvin AM, Garg A, Griner SB, Moore JD, Thompson EL (2022). Health literacy correlates to HPV Vaccination among US adults Ages 27–45. J Cancer Educ.

[87] WHO. Ten health issues WHO will tackle this year. 2019. https://www.who.int/news-room/spotlight/ten-threats-to-global-health-in-2019. Accessed 2 Apr 2023.

[88] Faye SLB, Krumkamp R, Doumbia S, Tounkara M, Strauss R, Ouedraogo HG (2022). Factors influencing hesitancy towards adult and child COVID-19 vaccines in rural and urban West Africa: a cross-sectional study. BMJ Open.

[89] Lopez et al. HPV knowledge and vaccine acceptance among European adolescents and their parents: a systematic literature review. Public Health Rev. 2020. https://www.ncbi.nlm.nih.gov/pmc/articles/PMC7222509/. Accessed 18 Dec 2022. 10.1186/s40985-020-00126-5 PMC7222509 32435520

[90] Layland EK, Carter JA, Perry NS, Cienfuegos-Szalay J, Nelson KM, Bonner CP (2020). A systematic review of stigma in sexual and gender minority health interventions. Transl Behav Med.

[91] Stanley M (2014). HPV vaccination in boys and men. Hum Vaccines Immunother.

[92] Takla A, Wiese-Posselt M, Harder T, Meerpohl JJ, Röbl-Mathieu M, Terhardt M (2018). Background paper for the recommendation of HPV vaccination for boys in Germany. Bundesgesundheitsblatt Gesundheitsforschung Gesundheitsschutz.

[93] WHO. Guide to introducing HPV vaccine into national immunnization programmes. 2016.

[94] Toh ZQ, Russell FM, Garland SM, Mulholland EK, Patton G, Licciardi PV (2021). Human papillomavirus vaccination after COVID-19. JNCI Cancer Spectr.

[95] Lam E. Vaccine-preventable diseases in humanitarian emergencies among refugee and internally-displaced populations. Human Vaccines & Immunotherapeutics. 2015. https://www.tandfonline.com/doi/full/10.1080/21645515.2015.1096457. Accessed 25 Nov 2022. 10.1080/21645515.2015.1096457 PMC4685677 26406333

[96] Jain M. Use of community engagement interventions to improve child immunisation in low- and middle‐income countries: A systematic review and meta‐analysis. 2022. https://onlinelibrary.wiley.com/doi/10.1002/cl2.1253. Accessed 25 Nov 2022. 10.1002/cl2.1253 PMC9359116 36913200

[97] Farooq et al. F. COVID-19 Vaccination and the Challenge of Infodemic and Disinformation. 2021. https://jkms.org/DOIx.php?id=10.3346/jkms.2021.36.e78. Accessed 25 Nov 2022. 10.3346/jkms.2021.36.e78 PMC7961870 33724740

[98] Rzymski P, Borkowski L, Drąg M, Flisiak R, Jemielity J, Krajewski J (2021). The strategies to support the COVID-19 vaccination with evidence-based communication and tackling misinformation. Vaccines.

[99] Kakisina PA, Indhiarti TR, Al Fajri MS (2022). Discursive strategies of Manipulation in COVID-19 political discourse: the case of Donald Trump and Jair Bolsonaro. SAGE Open.

