# Supplementary material for: Barriers and facilitators of HPV vaccination in sub-saharan Africa: a systematic review
Source: BMC Public Health. 2023 May 26;23:974. doi: 10.1186/s12889-023-15842-1 (PMC10214362; doi:10.1186/s12889-023-15842-1)
Supplement: Supplementary file 1 — Supplementary Material 1 [file 12889_2023_15842_MOESM1_ESM.docx]

**Supplementary Table 1**: study quality quantitative studies

Answer categories: Y = YES, N = NO, U = unclear; N/A = not applicable

| **Author, date** | **1. Was the sample frame appropriate to address the target population?** | **2.  Were study participants sampled in an appropriate way?** | **3.  Was the sample size adequate?** | **4. Were the study subjects and the setting described in detail?** | **5. Was the data analysis conducted with sufficient coverage of the identified sample?** | **6. Were valid methods used for the identification of the condition?** | **7.  Was the condition measured in a standard, reliable way for all participants?** | **8.  Was there appropriate statistical analysis?** | **9. Was the response rate adequate, and if not, was the low response rate managed appropriately?** | **Overall appraisal (include / exclude)** |
| --- | --- | --- | --- | --- | --- | --- | --- | --- | --- | --- |
| **Enebe et. al. (2021)** | **Y** | **Y** | **Y** | **Y** | **U** | **Y** | **Y** | **Y** | **Y** | **(7 Y) include** |
| **Li et. al. (2022)** | **Y** | **Y** | **N** | **Y** | **Y** | **Y** | **U** | **Y** | **U** | **(6 Y) include** |
| **Kassa et. al. (2021)** | **Y** | **Y** | **Y** | **Y** | **Y** | **Y** | **Y** | **Y** | **Y** | **(9 Y) include** |
| **Muhwezi et. al. (2014)** | **Y** | **Y** | **Y** | **N** | **Y** | **Y** | **U** | **Y** | **U** | **(6 Y) include** |
| **Ebu et al. (2021)** | **Y** | **Y** | **Y** | **Y** | **Y** | **Y** | **N** | **Y** | **U** | **(7 Y) include** |
| **Garon et. al. (2022)** | **Y** | **Y** | **U** | **N** | **U** | **Y** | **Y** | **Y** | **U** | **(5 Y) include** |
| **Asare et. al. (2020)** | **Y** | **Y** | **Y** | **Y** | **Y** | **Y** | **Y** | **Y** | **U** | **(8 Y) include** |
| **Ezenwa et. al. (2013)** | **Y** | **Y** | **Y** | **U** | **Y** | **Y** | **U** | **Y** | **U** | **(6 Y) include** |
| **Mabeya et. al. (2021)** | **Y** | **Y** | **U** | **Y** | **U** | **Y** | **Y** | **Y** | **Y** | **(7 Y) include** |
| **Milondzo et. al (2021)** | **Y** | **Y** | **U** | **N** | **U** | **Y** | **Y** | **Y** | **Y** | **(6 Y) include** |
| **Massey et. Al. (2017)** | **Y** | **Y** | **U** | **Y** | **U** | **Y** | **Y** | **Y** | **Y** | **(7 Y) include** |
| **Poole et. Al. (2013** | **Y** | **Y** | **N** | **Y** | **U** | **Y** | **Y** | **Y** | **Y** | **(7 Y) include** |
