# Supplementary material for: Barriers and facilitators of HPV vaccination in sub-saharan Africa: a systematic review
Source: BMC Public Health. 2023 May 26;23:974. doi: 10.1186/s12889-023-15842-1 (PMC10214362; doi:10.1186/s12889-023-15842-1)
Supplement: Supplementary file 2 — Supplementary Material 2 [file 12889_2023_15842_MOESM2_ESM.docx]

**Supplementary Table 2**: study quality qualitative studies

Answer categories: Y = YES, N = NO, U = unclear; N/A = not applicable

| **Author, date** | **1. Was the sample frame appropriate to address the target population?** | **2.  Were study participants sampled in an appropriate way?** | **3.  Was the sample size adequate?** | **4. Were the study subjects and the setting described in detail?** | **5. Was the data analysis conducted with sufficient coverage of the identified sample?** | **6. Were valid methods used for the identification of the condition?** | **7.  Was the condition measured in a standard, reliable way for all participants?** | **8.  Was there appropriate statistical analysis?** | **9. Was the response rate adequate, and if not, was the low response rate managed appropriately?** | **Overall appraisal (include / exclude)** |
| --- | --- | --- | --- | --- | --- | --- | --- | --- | --- | --- |
| **Remes et al. (2012)** | **Y** | **Y** | **Y** | **Y** | **Y** | **Y** | **Y** | **Y** | **Y** | **(9 Y) include** |
| **Vermandere et al. (2014)** | **Y** | **Y** | **U** | **Y** | **Y** | **Y** | **Y** | **Y** | **Y** | **(8 Y) include** |
| **Ports et al. (2013)** | **Y** | **Y** | **U** | **Y** | **Y** | **Y** | **Y** | **Y** | **Y** | **(8 Y) include** |
| **Nabirye et al. (2020)** | **Y** | **Y** | **Y** | **Y** | **Y** | **Y** | **Y** | **Y** | **Y** | **(9 Y) include** |
| **Massey et al. (2017)** | **Y** | **Y** | **Y** | **N** | **Y** | **Y** | **U** | **Y** | **U** | **(6 Y) include** |
| **Delany-Moretlwe et. al. (2018)** | **Y** | **Y** | **Y** | **Y** | **U** | **Y** | **U** | **Y** | ***not applicable*** | **(6 Y) include** |
| **Vermandere et. al. (2015)** | **Y** | **U** | **U** | **Y** | **Y** | **Y** | **Y** | **Y** | **Y** | **(7 Y) include** |
| **Kisaakye et. al. (2018)** | **Y** | **Y** | **U** | **N** | **Y** | **Y** | **Y** | **Y** | **Y** | **(7 Y) include** |
| **Turiho et. al. (2017)** | **Y** | **Y** | **Y** | **Y** | **Y** | **Y** | **Y** | **Y** | **Y** | **(9 Y) include** |
