# Supplementary material for: Barriers and facilitators of HPV vaccination in sub-saharan Africa: a systematic review
Source: BMC Public Health. 2023 May 26;23:974. doi: 10.1186/s12889-023-15842-1 (PMC10214362; doi:10.1186/s12889-023-15842-1)
Supplement: Supplementary file 3 — Supplementary Material 3 [file 12889_2023_15842_MOESM3_ESM.docx]

**Supplementary Table 3**: Characteristics of the selected quantitative studies

Abbreviations:

*= were appraised both in quantitative and qualitative studies

| **Authors** | **Study Design** | **Country** | **Instrument** | **Target Group** | **Sample**  **Characteristics** | **Age** | **Outcome Measures** | **Barriers and facilitators** | **Strengths (S)**  **Limitations (L)** |
| --- | --- | --- | --- | --- | --- | --- | --- | --- | --- |
| Enebe et. Al. (2021) [43] | Cross-Sectional | Nigeria | Structured, interviewer- administered, pretested questionnaire | Public & private female secondary school teachers  Not specifically gender centred | N= 377,  Tertiary education (69%), mean teaching duration 10.09 years, 75.3% engaging in other form of jobs next to teaching | Mean:  37.46 years  Mode:  30-39 years | Pearson’s Chi-square, Student’s T-test | 3.4% of respondents have been vaccinated, 5.6% of children were vaccinated. Most common reasons for non-administration:  - **Non-availability** (46.31%)  - **High Costs of HPV vaccines** (25.12%)  Among the vaccinated, 81% vaccinated their female children and 19% both male and female children. No male child was vaccinated on its own.  **Previous history of vaccination** of respondents significantly predicted the vaccination of their children and relatives (AOR = 6.069), the more monthly income, the higher the likelihood of children being vaccinated by respondents, significant association between **uptake of vaccines and address of HPV vaccines** in schools  Conclusion:  Overall knowledge of HPV is low, but acceptance high. The introduction of cervical cancer vaccination education of the teachers in the secondary schools will improve the uptake among adolescent´s in the country. | (S):  Multistage sampling of participating schools, good description of sample size calculation and sampling method  (L): Inclusion of just female secondary school teachers. Associations just calculated via Chi-Square |
| Li et. Al. (2022) [56] | Cross-  Sectional | Tanzania | Piloted, in- person tablet-based surveys | Health workers, school personnel, community leaders  Female centred | N= 461 (165 health worker: 14 % male, 86% female, 135 school personnel: 21% male, 79% female, 143 community leaders: 50 percent male, 50% female, 18 council leaders: 67% male, 33% female), | Age Range: Under 25- 55 and over | Descriptive statistic | Most girls receive HPV vaccine in primary school (stated by 51% of HCW) 48% of school personal indicated they felt HPV vaccine not well integrated in routine immunization  **Misinformation** heard regarding the vaccine is stated by 54% HCW, 57% school personnel, 43% Community leaders and 61% of council leaders (98,96,98,91% affecting the girl’s fertility), respondents say they do **not have sufficient mobilization/communication material** (44-92%)  Due to COVID-19, vaccination was interrupted due to closure of schools for several months, responding to this, Tanzania implemented a recovery plan including communication with the ministry of education to facilitate vaccination in schools as well as social mobilization to communities and schools and production of TV and radio spots.  Conclusion:  The Tanzanian example provides examples of vaccine delivery to an older cohort as well as the feasibility and challenges of the approach. | (S):  Variety of viewpoints in sample, detailed description of sampling technique, piloted and country adapted questionnaire, limitations of the study cleary explained  (L):  Only descriptive statistics offered, even though there is potential for further analysis, strong variability in the items per occupation group, not nationally representative |
| Kassa et. Al. (2021) [42] | Cross-Sectional | Ethiopia | Structured, self-administered questionnaire | Female students enrolled in the Minjar-shenkora district in 2020  Female centred | N= 591 Students,  Most students (81.2%) grade 7 & 8  78% of respondents from rural districts | 12.5% Age 11 to 13  87.5 % Age 14 and 15 | Binary & multiple logistic regression and 95% Cis | Primary source of information about HPV are health professionals (60.1%), 66.5% (CI 0.63-0.7) of the participants ever received an HPV vaccine  Factors associated with practice of vaccination:   - **Being knowledgeable about HPV vaccine** (AOR= 8.65 [CI 5.2- 14.3]) - Positive attitude towards HPV vaccination (AOR= 1.85 [CI 1.18-3])   In comparison to urban areas, students from **rural areas** were 88% less likely to practice HPV vaccine (AOR = 0.12 [CI 0.07-0.21])  Conclusion:  Health facilities should include routine strategies for vaccine delivery in addition to the campaign-based delivery to increase the uptake of HPV vaccination, especially in rural areas. Schools and health professionals as focal points should strengthen knowledge building strategies for students. | (S):  Detailed description of multi-stage sampling technique, holistic statistical analysis  (L):  Enrollment of just female students,  Displayed tables and figures not intuitive  Small proportion of participant in the lower age group |
| Muhwezi et. Al. (2014) [44] | Cross- Sectional | Uganda | Pre-coded self-administered questionnaire | Parents of secondary school boys from districts where a vaccination of girls took place compared to parents from districts where no vaccination took place  Male centered | Intervention:  N= 456  Control: N= 414 | Intervention:  Mean age 40.8  (SD =15)  Control:  Mean Age 40.6  (SD= 12.7) | Pearson’s Chi-square, Student’s T-test, Binary logistic regression, Odds Ratios with 95% Cis | 78.3% of parents willing to give their sons HPV vaccination in the future, no difference in willingness to vaccinate sons was seen in the different districts.  Parents who agreed to the statement of their son being at **high risk to contract HPV** were more likely to show willingness to allow HPV vaccination (35% vs. 17.3%, Crude OR: 2.72, CI: 1.24–5.95, ꭓ^2^= 6.63, p = 0.010).  Parents who knew that HPV is sexually transmitted (81.1% vs. 65.8%, Crude OR: 2.23, CI: 1.31–3.80, ꭓ^2^= 9.17, p= 0.002), those who knew that males could acquire HPV (Crude OR: 3.10, CI: 1.79–5.42, ꭓ^2^= 18.37, p= 0.000), and that the HPV vaccine effectively protects against HPV (Crude OR: 2.33, CI: 1.42–3.81, ꭓ^2^= 12.19, p= 0.000) are showing significantly more willingness to allow their son to receive HPV vaccines in the future.  Acceptance of HPV vaccination of daughters (AOR= 9.97; CI: 4.57–21.76) and being likely to recommend the HPV vaccine to son(s) of friends and relatives (AOR= 18.25; CI: 8.32–40.04, p= 0.000) predicted parental willingness to allow son to give the HPV vaccine in future  Conclusion:  Future attempts to vaccinate males against HPV in Uganda should concentrate on the link of HPV infection, men’s health in general and sexual health. It is necessary to promote confidence in health workers for parents to accept HPV vaccines for their male children. | (S):  Including male participants, detailed description of pre-tested investigation tool translated into local dialects,  Comprehensive discussion of strengths and limitations  (L):  Responses of parents’ hypothetical as vaccine is not introduced in the country yet |
| Ebu et al. (2021) [45] | Cross-Sectional | Ghana | Adapted questionnaire from previous studies | Female, state registered nursing certificate or diploma midwifes and nurses  Female centred | N= 318,  45% married, 48.1% diploma & 37.1% bachelor’s degree | 47.5% Age group 20-29,  37.1% 30-39, 9.1% 40-49,  6.3% 50-59 | Chi- Square, Fishers- exact test, scoring of knowledge | 41.5% (n = 132) of the participants had high levels of knowledge of cervical cancer risk factors, and 17.6% (n = 56) of the respondents had received at least one dose of the HPV vaccine.  Reasons for accepting HPV vaccine:   - Advice from a colleague (12.9%) - Perceived threat of cervical cancer (11.7%)   Out of the non-vaccinated respondents (n= 262):   - 24.45% strongly agreed and 28% agreed having **limited information** on HPV vaccine   Significant associations with acceptance of HPV and age (ꭓ^2^= 23.746, p= 0.001), marital status (ꭓ^2^= 14.758, p= 0.005), completed **level of education** (ꭓ^2^= 21.692, p= 0.001) and **duration of working at the hospital** (ꭓ^2^= 8.424, p= 0.038).  Conclusion:   - Need for targeted knowledge and attitude improvement - These interventions should consider sociodemographic characteristics of the nurses and midwifes targeted | (S):  Pretested study instrument,  (L):  All female study sample, sampling located at one study site, convenience sampling approach poses risk of selection bias |
| Garon et. Al. (2022) [41] | Two stage cluster sampling with 2 cross-sectional surveys  in 2018 and 2019 | Zimbabwe | Standard, tablet-based questionnaires, administered by interviewers | Health workers, school personnel and community members  Female centered | N= 221  56 health care workers, 55 school health coordinators, 55 village health workers, 55 community leaders  Healthcare workers 75% female, school health coordinators 62% female, village health workers 84% female, community leaders 18% female | Age range: 25- 55 and over  Mode: HCW, health coordinators, village health workers: 45-54,  community leaders: 55 and over | Descriptive Analysis | Understanding of HPV vaccine for cervical cancer prevention (96% HCW, 98% school health coordinators) and the infection of HPV is high.  66% reported to have enough staff to support vaccinations in schools, 61% reported to have enough staff to maintain routine immunization services in health facilities.75% of health worker indicated their workload as “somewhat or greatly increased”.  Main challenges identified overall:   - **Inadequate social mobilization or materials** (28%) - **Insufficient transportation of staff and supplies** (26%) - **Inadequate training** (18%)   Main challenges identified by the health workers:   - **Lack of funds for delivering the vaccine** (34%) - **Shortage to conduct vaccine sessions** (25%)   Common rumors**/ misinformation** identified:   - “HPV vaccine will affect a girl’s fertility” - “HPV vaccine is not safe” - “HPV vaccine is experimental” - “HPV vaccine will cause severe side effects”   **Knowledge of the correct target cohort** eligibility decreased from 91% in 2018 to 50% in 2020 among  health workers.  Conclusion:  HPV vaccine introduction showed feasible for the health system to implement and well accepted. Challenges identified are the targeting of eligible participants, potentially missing opportunities for vaccination. | (S):  Questionnaires possible to administer in local languages, discussion under consideration of Zimbabwe’s context  (L):  Insufficient description of sampling strategy and sample, only descriptive data analysis |
| Asare et. Al. (2020) [46] | Cross-Sectional | Ghana | Reliability tested, modified version of multi-theory model (MTM) questionnaire | Adolescents  Not specifically gender centered | N= 285  91.2% female  8.8% male  100% insured  84.9% did not have a primary health care doctor | Age range 12-17  Mean: 15.47 (±1.80) | Use of multi theory models to predict initiation and completion of HPV vaccine series,  Linear regression | 95.4% never heard of HPV vaccination or about HPV (92.3%). Participatory dialogue of HPV vaccination (M = 2.98, SD = 5.30) was very low.  **Female participants are more likely to get the first dose of vaccination compared to males** (M= 3.46 vs. M= 2.64, p < 0.01) and females are **more likely to complete** the recommended doses of vaccines (M= 3.78 vs. M= 1.64, p < 0.001).  Perceived beliefs (R^2^ = 6.1%) and change in physical environment (R^2^ = 8.8%) are predictors of the adolescent’s likelihood of getting the first dose of HPV vaccination (p < 0.001).  Perceived beliefs (R^2^ = 7.8%), practice for change subscale (R^2^ = 8.1%), and emotional transformation subscale (R^2^ = 1.1%) are predictors of the likelihood of completing the recommended series of HPV vaccination (p < 0.001).  Conclusion:  Findings are showing the lack of awareness of HPV related cancers in Ghana. Future interventions should consider the modifiable factors identified to increase adolescents HPV vaccination uptake and completion. | (S):  Comprehensive statistical data analysis,  Reliability tested questionnaire,  Comprehensive discussion with view on strengths and limitations of the study, adequate sample size  (L):  Response rate not described,  Convenience sample might impose risk of selection bias |

| Ezenwa et. Al.  (2013) [47] | Cross-Sectional | Nigeria | | structured, pretested, interviewer-administered questionnaire | | Mothers of female adolescents  Female centred | | N= 290  1-6 female daughters (mean 2.0 ± 1.0) | Age range 24–62 years  Mean: 40.1 (±6.9) | | Descriptive analysis for the identification of barriers to vaccination,  Chi-square | Low awareness of HPV (27.9%) and HPV vaccines (19.7%) among the mothers even though awareness on cervical cancer is high. As the source of information, 32% of the women indicated the media. Main reason of not willing to vaccinate is indicated with inadequate information  Awareness and utilization of HPV vaccines increased with higher educational level (ꭓ2= 61.07, p= <0.001).  Identified barriers of vaccination among mothers:   - Vaccine is too costly (22.8%) - Access to vaccines (51.5%) - Insufficient information (11.8%)   Conclusion:  Knowledge and awareness of HPV and HPV vaccines in Nigeria is low. Despite low knowledge, mothers are willing to vaccinate their daughters. Knowledge improvement through education and possible inclusion of the vaccine in the national immunization schedule is recommended to eliminate financial barrier. | | | | | (S):  Recommendations to implement HPV vaccination into existing vaccination visits for adolescents in Nigeria,  (L):  Short description of methods, more extensive description of recruitment is needed. All female sample | | |
| --- | --- | --- | --- | --- | --- | --- | --- | --- | --- | --- | --- | --- | --- | --- | --- | --- | --- | --- | --- |
| Mabeya et. Al.  (2021) [55] | Cross-Sectional | Kenya | | Validity tested, semi-structured questionnaires | Mothers of female adolescents  Female centred | | N= 300  85% secondary/ tertiary education  78% married | | Mean: 34 years | | descriptive and inferential statistics  (OR) | | | Vaccination against HPV low at (9.4%), 85% are aware of cervical cancer, 60% of HPV as well as 62% of the vaccine. 30% are not willing to accept the HPV vaccine.  Information sources:   - Health personal (39%) - Workshop/ conference (30%)   Reasons for vaccination:   - **Fear of cervical cancer** (90.5%) - **Routine immunization** (20.75%) - **To prevent STD** (29.6%) - **Life experience with cervical cancer** (17.3%)   Reasons for non-vaccination:   - **High costs of HPV vaccine** (56.9%) - **Concerned for side effects** (60.8%) - **Non availability** (64.7%) - **Fear of promoting sexual promiscuity** (90.5%)   Conclusion:  Awareness of HPV and the vaccine is still suboptimal among mothers of adolescent daughters in Kenya. Implementation into the routine immunization schedule, as well as policy building strategies to provide a legal framework for service users, NGOs and activists is recommended. | | (S):  Validity pretested instrument, Holistic description and discussion of barriers of vaccination  (L):  All female sample, description of data analysis unclear, | | |  |
| Milondzo et. Al  (2021) [58] | Cross-Sectional | South Africa | Pre-tested online questionnaire | | caregivers of girls in private school schools aged ≥9 years  Female centered | | N= 615  91.4% female,  88.1% biological parents of the girl,  Respondents who completed the test were older, more educated, and more likely to be the biological parent of the girl | | 44.7% [44.7-52.9] age group 40-49 | Score categorization for knowledge, attitude including descriptive statistics, frequency distribution (95% CI) of willingness to vaccinate, inferential data analysis measuring associations between knowledge, coverage, attitudes including OR (95% CI) | | | Caregivers are showing good knowledge in 76.5% and positive attitude in 45.3% towards HPV vaccination. The daughters have already received the vaccine in 19.4% of the cases.  Out of the unvaccinated girls, caregivers were more likely **influenced by sources like online articles or vaccine injury reports** than caregivers of vaccinated girls (OR= 3.8 [CI: 2.1–6.9]) and more likely to be influenced by **alternative medical practitioners** (OR= 0.2 [CI 0.05-0.9]).  Caregivers of vaccinated girls are more likely have **access to HPV vaccination information** (OR: 2.0; 95% CI: 1.23.4; p = 0.006) and more likely to base their **vaccination decision on the advice of healthcare providers** (OR:3.6 [CI: 2.1–6.1])  Negative attitude toward HPV vaccination predicted non-vaccination of daughters (OR: 0.2 [ CI: 0.1–0.3]). Caregivers with **good knowledge** scores are more likely to have positive attitudes towards vaccination than those with poor knowledge scores (OR: 2.8 [CI: 1.7–4.7])  No significant difference is seen in medical insurance coverage.  Conclusion:  Misinformation is identified as main driver of negative vaccination attitudes, resulting in low vaccination rates, indicating that free school-based HPV vaccination may not increase coverage to an optimal level. Advocacy targeting stakeholders is recommended. | | (S):  Inclusion of female and male caregivers,  Cognitive pretested Study instrument  Thoroughly described score calculation  Extensive discussion of results with display of strengths and limitations of the study  (L):  Data collection via email contact and Facebook adverts might impose a selection bias, no possibility to verify the relationship status to the child via the online survey | | |  |  |
| Massey et. Al.  (2017) [50]***** | Cross-Sectional | Senegal | Self-administered questionnaire based on formative research and adapted from the Senegal DHS survey | | Adolescents and young adults  Not specifically gender centred | | N= 2286,  80% Muslim  56% female  44.8% male  29% rating themselves as in excellent health | | Age range 14-22 | Chi-Square, Multivariable logistic regression | | | Of the participants, 27% have heard of HPV, out of these, 28% indicated willingness to vaccinate.  **Participants from rural areas had 63% higher odds (95% CI: 1.24, 2.12) of having heard of HPV than those in urban areas.**  While participants with a father that completed **higher education** had a 41% higher chance (95% CI: 1.16- 1.92, p <0.05) of being aware of HPV, the increasing level of education of the father is negatively associated with the willingness to vaccinate.  Students that previously spoke to a health care professional about the HPV vaccine have 80% higher chances (95% CI: 1.116-2.81) to be willing to accept vaccination.  Conclusion:  Due to the low level of awareness, the crafting of effective public health communication such as health messages and information is recommended in combination with media efforts. Integration into the routine immunization schedule is likely to facilitate vaccine uptake. | | (S):  Adequate sample size  Sufficient statistical analysis  Analysis of the target group young adolescents  (L):  Self-administered questionnaire, if the person is not speaking French the study is orally administered  Age range is older than the prioritized vaccination age | | |  |  |
| Poole et. Al.  (2013) [59] | Cross-Sectional | Mali | Face to face structured interviews using a standardized questionnaire | | Adolescent and adult females and males  Not specifically gender centred | | N= 51  49% adolescent,  25 female & 26 male participants,  43.1% married | | Age range 12-17 and greater equal 18,  Mean age 26.1 (±14.6) | Descriptive analysis, Fishers exact test | | | Low knowledge of HPV is reported in the sample. Of the participants 54.9% know places of STI screening and treatment offering. Only 2% of the sample is aware that HPV is an STI.  Out of the participants, 24% thought that only females are affected by HPV and 30% thought only males are infected.  All participants stated that they would like to have the vaccination available in Mali and 74.5% would be willing to vaccinate their children.  **Males are showing greater autonomy** in the decision making whether to vaccinate or not compares with women and adolescents (OR: 0.176 [95%CI 0.03–0.61]; McNemar’s test p = 0.0026).  Conclusion:  Men are defined as the primary decision maker in a predominant Muslim population regarding the HPV vaccine. Autonomy in taking vaccine decision is not equally distributed. Frameworks for reproductive health therefore need to incorporate men into emerging immunization programs. | | (S):  Inclusion of male and female adolescents, standardized questionnaire, display of vaccination autonomy  (L):  Small sample size, insufficient explanation of statistical analysis | | |  |  |
