# Supplementary material for: Barriers and facilitators of HPV vaccination in sub-saharan Africa: a systematic review
Source: BMC Public Health. 2023 May 26;23:974. doi: 10.1186/s12889-023-15842-1 (PMC10214362; doi:10.1186/s12889-023-15842-1)
Supplement: Supplementary file 4 — Supplementary Material 4 [file 12889_2023_15842_MOESM4_ESM.docx]

**Supplementary Table 4**: Characteristics of the selected qualitative studies

*= were appraised both in quantitative and qualitative studies

| Authors | Study design | Country | Target Group | Sample Characteristics | Outcome measures | Barriers and facilitators | Strengths (S)  Limitations (L) |
| --- | --- | --- | --- | --- | --- | --- | --- |
| Remes et al. (2012 [48] | Cross-sectional | Tanzania | Parents, female pupils, teachers, health workers and religious leaders  Female centered | 169 participants  109 female participants  60 male participants | Knowledge and acceptability of vaccine, views on delivery and consent procedures. | Attitudes towards HPV vaccination:  Most respondents welcomed the HPV vaccine. Almost all would allow their daughter to be vaccinated.  Facilitators: Previous **positive vaccine experiences** (measles, tetanus or polio). **Adding** HPV vaccination to other health services. **Governmental approval** of vaccines. Vaccine hesitancy could be overcome by **appropriate information**. **Opt-out consent**.  Barriers: **workload of HCW** as missing **infrastructures and training**. **Low awareness** and no or **incorrect knowledge** of cervical cancer and HPV among all participants | (S): description of opt-out consent. Large variety of participants.  (L): small purposive samples and in schools, a teacher selected the parent, student and teacher participants for GDs who might have been the most accepting of new health interventions. |
| Vermandere et al. (2014) [53] | Cross-sectional | Kenya | Female participants only  Female centered | (n = 287) baseline  (n = 256) follow-up | baseline acceptance  follow-up acceptance  assessing practical barriers  correlation of information and vaccine acceptance rate | Baseline acceptance: 88.1%  Follow-up coverage: 31,1%  Barriers reported through the baseline: concerns about **side effects**, infertility, and unsafe administration of the vaccine. Doubts on the **efficacy** of the vaccine, as **missing partners approval** and concerns about the young age of the daughter.  Barriers reported through the uptake/follow-up:  **not receiving information** regarding where and when the vaccination took place was the most important barrier.  Fear of side effects. Opposition by partner, community, or daughter herself. | (S): even if the vaccine is accepted, the uptake is largely determined by obtaining appropriate and practical information about vaccination opportunities. Mentioned the weight of social influences on decision-making.  (L): uptake was more determined by program awareness than by HPV vaccine acceptance. |
| Ports et al. (2013) [60] | Cross-sectional | Malawi | Mothers of daughters  Female participants only  Female centered | n=30 mothers aged 18–49 years | Knowledge and beliefs about HPV, cervical cancer, and vaccination, and their social-ecological contexts were explored. | Barriers: **knowledge** limited on cervical cancer and missing completely for HPV vaccine.  Facilitator: **cervical cancer as a severe disease**, trust in health infrastructure, **health education** from HCW. Information dispersed throughout community and endorsed by the **government**. Positive experiences with vaccines in the past. Women as peer health leaders. | (S): encouraging women to become peer health leaders could be a key to successful vaccination programs  (L): small, potentially non-representative sample |
| Nabirye et al. (2020) [49] | Cross-sectional | Uganda | Female adolescents and District Health Officials  Female centered | 407 respondents, 6 key informant interviews  Female adolescents aged 9–15 years | vaccine uptake, barriers to vaccination. | Coverage: low uptake (14%). Target of 80% not reached. Uptake higher when associated with other (health)-services or previous conducted vaccination.  Barriers: **lack of awareness** on the HPV vaccines, **inadequate staff**,  Facilitators: having many delivery options or **clear target** as **school**-based delivery, getting **information on side effects**, **combining different health services** with the vaccine uptake  Health official barriers reported: missing (communicated) coverage target rate, inconsistency in vaccine supply and inadequate training. | (S): addressed key areas to enhance HPV Vaccine uptake in Uganda  (L): cross-sectional cannot infer a causal relationship between awareness of the vaccine and subsequent uptake. |
| M. Massey et al. (2017) [50]* | Cross-sectional | Senegal | Pupils/students  Not specifically gender centred | Participants from 6 high schools and 5 community centers across five regions of Senegal (urban, suburban, and rural regions) (n=2.286)  Age range 14-22 years  Female respondents represent 56% of the sample | vaccine awareness and receptivity, willingness to vaccinate among adolescents and young adults | Facilitators: Health care providers and parents are important stakeholders in disseminating HPV vaccine information. Frequent contact with health care providers can increase awareness.  Barriers: parents (especially father) **low education**  Awareness: study indicated low awareness of HPV and low willingness for vaccine uptake. | (L): sample age range of 14–22 years but target age is 9-13 years.  (S): balanced geographical distribution of the participants (rural/urban). |
| Delany-Moretlwe (2018) [51] | Cross-sectional | South-Africa | Campaign assessment  Female centred | Review of Records and Materials (as field guide, consent forms, invitation letters, data collection tools, and vaccination card)  Direct Observation of 7 Vaccination Sessions  29 Key Informant Interviews  Assessment of Media Coverage (900 print, and online media, 80 newspapers, 291 community publications, 95 magazines, 37 radio stations, and 13 television show) | Lessons learned from the implantation campaign: challenges, facilitators, and barriers to a successful roll-out. | Barriers / Challenges: **obtaining informed consent**, vulnerabilities in cold chain capacity (**poor logistics**), and onsite management of minor **adverse events**. Campaign planners do not anticipated misinformation about HPV vaccination on social media. Misunderstanding and complicated consent form needed to be signed by parents.  Facilitators: high-level political mandate and **high political commitment**, centralized organization, and planning. Targeting vaccination at schools. Highly skilled health care workers at all levels. Consideration of seasons (rain) and access to infrastructures. Managing of adverse events even if small as a big impact.  *Suggestion*: using opt-out consent / alternative strategies for seeking consent from parents. Tackle misinformation on social media. | (S): clear suggestions to improve HPV vaccination campaigns.  (L): limited sampling of key informants and purposeful sampling for the observation. Only 1 researcher was responsible for conducting the document review. |
| Vermandere et al. (2015) [53] | Cross-sectional | Kenya | Teachers, fathers, and vaccinators  Female centred | 30 men and 28 women | Assess awareness and attitudes towards the vaccination program, cervical cancer, and the HPV vaccine.  Discuss the course of the program and potential improvements. | Barriers: Cervical cancer was poorly understood by fathers and teachers and mainly linked with nonconforming sexual behaviour and modern lifestyle.  Facilitators: Close collaboration between teachers and health staff. | (S): targeting the understanding of fathers.  (L): not all teachers who participated in the FGD gave classes to girls in class from target group of the vaccination. FGD with fathers were not transcribed verbatim in Swahili but were simultaneously translated into English -> loss of culturally specific concepts. Possible recall bias. Lack of insight in how promotion was implemented. |
| Kisaakye et al. (2018) [54] | Cross-sectional | Uganda | Female adolescent  Female centred | 460 participants  and 5 key informants  female adolescent aged 12-17 years | Uptake (3 doses received), factors associated with uptake. | Facilitators: attaining ordinary level of **education**, **positive attitude** towards the vaccine, receiving vaccine doses from different vaccination, encouragement from health workers, community outreaches, availability of vaccines at vaccination sites and receiving full information about the vaccine  Barriers: uptake was low due to lacking consistent supply of vaccines at the vaccination sites and low health education as well as missing community outreaches. | (L): uptake associated with 3 vaccine doses is challenging. |
| Turiho et al. (2017) [52] | Cross-sectional | Uganda | Schoolgirls, parents/guardians, schoolteachers, health workers and community leaders.  Female centred | Girls aged 13–16 years | Perceptions about HPV vaccination. | Facilitators: understanding that cervical cancer is a **serious disease** and that HPV vaccination in combination with low side effects of vaccination is an effective protection.  Barriers: Misconceptions about **safety** of the HPV vaccination were largely based on **previous experiences of adverse effects** of other vaccinations. | (S): perspective on vaccinations in terms of a personal health cost/benefit ratio.  (L): recall bias possible, data collected 1 year after vaccination. Selection bias for FGD. |
